# Immunogenicity and protective efficacy of OmpA subunit vaccine against *Aeromonas hydrophila* infection in *Megalobrama amblycephala*: An effective alternative to the inactivated vaccine

**DOI:** 10.3389/fimmu.2023.1133742

**Published:** 2023-03-09

**Authors:** Minying Zhang, Ting Zhang, Yang He, Hujun Cui, Hong Li, Zehua Xu, Xu Wang, Yunlong Liu, Hongping Li, Xiaoheng Zhao, Hanliang Cheng, Jianhe Xu, Xiangning Chen, Zhujin Ding

**Affiliations:** ^1^ College of Marine Life and Fisheries, Jiangsu Key Laboratory of Marine Biotechnology, Jiangsu Key Laboratory of Marine Bioresources and Environment, Co-Innovation Center of Jiangsu Marine Bio-industry Technology, Jiangsu Ocean University, Lianyungang, China; ^2^ Key Laboratory of Sichuan Province for Fishes Conservation and Utilization in the Upper Reaches of the Yangtze River, Neijiang Normal University, Neijiang, China; ^3^ Jiangsu Institute of Marine Resources Development, Lianyungang, China; ^4^ Hunan Fisheries Science Institute, Changsha, China

**Keywords:** *Aeromonas hydrophila*, inactivated vaccine, OmpA subunit vaccine, immunogenicity, Immunoprotection

## Abstract

*Aeromonas hydrophila* is a kind of zoonotic pathogen, which can cause bacterial septicemia in fish and bring huge economic losses to global aquaculture. Outer membrane proteins (Omps) are conserved antigens of *Aeromonas hydrophila*, which can be developed as subunit vaccines. To evaluate the protective efficacy of inactivated vaccine and recombinant outer membrane protein A (OmpA) subunit vaccine against *A. hydrophila* in juvenile *Megalobrama amblycephala*, the present study investigated the immunogenicity and protective effects of both vaccines, as well as the non-specific and specific immune response of *M. amblycephala*. Compared with the non-vaccinated group, both inactivated and OmpA subunit vaccines improved the survival rate of *M. amblycephala* upon infection. The protective effects of OmpA vaccine groups were better than that of the inactivated vaccine groups, which should be attributed to the reduced bacterial load and enhanced host immunity in the vaccinated fish. ELISA assay showed that the titer of serum immunoglobulin M (IgM) specific to *A. hydrophila* up-regulated significantly in the OmpA subunit vaccine groups at 14 d post infection (dpi), which should contribute to better immune protective effects. In addition, vaccination enhanced host bactericidal abilities might also attribute to the regulation of the activities of hepatic and serum antimicrobial enzymes. Moreover, the expression of immune-related genes (*SAA, iNOS, IL-1 β, IL-6, IL-10, TNF α, C3, MHC I, MHC II, CD4, CD8, TCR α, IgM, IgD* and *IgZ*) increased in all groups post infection, which was more significant in the vaccinated groups. Furthermore, the number of immunopositive cells exhibiting different epitopes (CD8, IgM, IgD and IgZ) that were detected by immunohistochemical assay had increased in the vaccinated groups post infection. These results show that vaccination effectively stimulated host immune response (especially OmpA vaccine groups). In conclusion, these results indicated that both the inactivated vaccine and OmpA subunit vaccine could protect juvenile *M. amblycephala* against *A. hydrophila* infection, of which OmpA subunit vaccine provided more effective immune protection and can be used as an ideal candidate for the *A. hydrophila* vaccine.

## 1 Introduction

*Aeromonas hydrophila* is a kind of gram-negative bacteria with strong pathogenicity, which can infect human, livestock and aquatic animals. It is easy to proliferate and cause disease outbreaks in the aquaculture ponds during high temperature seasons, causing huge economic losses to the global aquaculture industry (1). *A. hydrophila* exhibits a wide range of pathogenicity, especially the explosive septicemia that has been found in most freshwater fish, such as *Megalobrama amblycephala*, *Hypophthalmichthys molitrix*, *Ctenopharyngodon idella*, *Cyprinus carpio* and the like, which exhibits the typical pathological features of surface hyperemia, anal redness and swelling with hemolysis (2). Its pathogenic factors mainly include exotoxins, extracellular enzymes, outer membrane proteins (Omps), S-layer proteins, and lipopolysaccharides (3). *A. hydrophila* commonly infects animals *via* adhesion and enterotoxic mechanisms, proliferating in the intestines and migrating to other tissues through blood circulation, and then synthesizing and secreting exotoxins, causing tissue lesions, systemic symptoms and death. At present, antibiotics are usually used to prevent and treat bacterial septicemia, but long-term use of antibiotics will not only cause antimicrobial resistance, but also affect the safety of aquatic products. Vaccine immunization is an important and environmentally effective way for disease prevention and control in aquaculture, and the significance of vaccine development and application is more prominent under the background of antibiotics reduction and substitution. So far, many studies focus on *A. hydrophila* vaccines, including whole bacteria inactivated vaccine (4), subunit vaccine (5), attenuated live vaccine (6), nucleic acid vaccine (7), etc., and has gone through the developmental stages from univalent vaccine to multivalent vaccine (8, 9).

Previous studies have found that the inactivated *A. hydrophila* vaccines provide relatively excellent immune protective effects on aquatic animals by inducing host immune responses. For instance, the inactivated *A. hydrophila* vaccine shows a significant immune protective effect on juvenile *Ictalurus punctatus*, with the specific antibody that has been induced and produced post immunization (4). Similarly, vaccination with inactivated *A. hydrophila* vaccine by intraperitoneal injection or immersion shows significant up-regulation of the skin mucus lysozyme and specific antibody levels in the serum and skin of *Piaractus mesopotamicus*, thereby increasing the survival rate post infection (10). Although attenuated live vaccines and nucleic acid vaccines of *A. hydrophila* also exhibit pretty good immune protective effects, these researches still remain in the laboratory stage (11–13).

However, due to the numerous serotypes of *A. hydrophila* and the wide range of pathogenic objects, it is difficult for inactivated vaccines to achieve broad-spectrum immune protection against *A. hydrophila* from different sources or serotypes. The various Omps of *A. hydrophila* with high immunogenicity are noteworthy (14), which can stimulate the humoral and cellular immunity of fish. Moreover, Omps are highly conserved among different strains (15), and are ideal antigens for preparing *A. hydrophila* subunit vaccines with broad-spectrum immune protective effects. The OmpA is a conserved antigen, which maintains the integrity of the outer membrane of *A. hydrophila*. The prepared OmpA antiserum of *A. hydrophila* J-1 strain isolated from *Carassius auratus* is broad-spectrum, which shows strong immunoreaction with nine *A. hydrophila* strains of different serotypes (16). In addition, the OmpA subunit vaccine of *A. hydrophila* also shows excellent immune protective effects on *I. punctatus* (17).

Therefore, the inactivated vaccines prepared by formalin inactivation and heat inactivation always exhibit excellent immune protective effects against specific *A. hydrophila* strain, but the broad-spectrum protective effects against various serotypes are limited. Usually, the protective effects and duration of protection of injection immunization are higher than that of immersion immunization for inactivated vaccines (13, 18). On the other hand, subunit vaccines prepared by isolation or recombinant expression of conserved antigens, usually show broad-spectrum immune protective effects against different serotypes of *A. hydrophila*. As the immunogenicity and stability of subunit vaccines are usually weaker than that of other types of vaccines, so injection immunization is a better choice to ensure excellent immune protective effects (19, 20).

In the present study, we evaluated the protective effects of inactivated and OmpA subunit vaccines against *A. hydrophila* infection in juvenile *M. amblycephala*, and clarified whether the concentration of vaccines affected the immune protection. In addition, the immunity of *M. amblycephala* upon infection was detected by measuring the density of immune cells, the expression of immune genes, the titer of serum antibodies and the bactericidal ability. This study provides novel insights into the comparative evaluation of immunogenicity and protective effects of *A. hydrophila* inactivated vaccine and OmpA subunit vaccine, so as the theoretical basis for developing high-efficiency aquatic vaccines.

## 2 Materials and methods

### 2.1 Ethics statement

This study was approved by the Animal Care and Use Committee of Jiangsu Ocean University (protocol no. 2020-37, approval date: September 1, 2019). All procedures involving animals were performed in accordance with the guidelines for the Care and Use of Laboratory Animals in China.

### 2.2 *A. hydrophila* strain

The pathogenic *A. hydrophila* strain isolated from moribund *M. amblycephala* was stored at -80°C in our laboratory, which had been used in our previous studies (21). The stored *A. hydrophila* strain was cultured on Luria-Bertani (LB) agar plates, and single colonies of *A. hydrophila* were randomly isolated for expanding culture at 28°C for 16 h, which were then identified by PCR amplification and *16S rRNA* sequencing (MAP, Shanghai, China). Then, the correctly identified *A. hydrophila* was stored at 4°C for further use.

### 2.3 Experimental fish

Healthy juvenile *M. amblycephala* (1.12 ± 0.22 g), obtained from a fish farm in Guangzhou, China, were confirmed free of infection by examining with standard bacteriological and parasitological examination (22). Experimental fish were fed with commercial feed for temporary rearing and taming for 2 weeks in an indoor freshwater recirculating system before the culture experiment.

### 2.4 Preparation of vaccines

#### 2.4.1 *A. hydrophila* OmpA subunit vaccine

According to the genomic DNA sequence of *A. hydrophila OmpA* gene (GenBank accession number: AF146597), the primers were designed to construct the recombinant plasmid including the coding region of *OmpA* gene (**Supplemental Table 1**). The PCR amplified product was digested and cloned into pET-32a vector, and then transformed into *Escherichia coli* BL21 competent cells (TransGen Biotech, Beijing, China). The recombinant OmpA protein was induced by isopropyl-β-D-thiogalactopyranoside (IPTG) with a final concentration of 0.5 mM at 37°C for 10 h, which was purified using a Ni-Agarose His-tagged Protein Purification Kit (CoWin Biosciences, Jiangsu, China). Then, the protein concentration was detected by BCA Protein Assay Kit (CoWin Biosciences).

#### 2.4.2 *A. hydrophila* inactivated vaccine

The identified *A. hydrophila* was incubated in LB liquid medium at 28°C for 24 h, followed by inactivated with 0.3% formaldehyde at 37°C for 16 h (23). Sterility assay was carried out by conventional plate culture method to ensure the safety of the prepared inactivated vaccine. Then, the inactivated *A. hydrophila* was collected by centrifuging at 3, 000 rpm for 10 min, and washed with phosphate buffered saline (PBS) for three times.

#### 2.4.3 Safety detection of prepared vaccines

The *A. hydrophila* OmpA subunit vaccine and inactivated vaccine was mixed with MONTANIDE™ ISA 763A VG adjuvant (Seppic, France) in the ratio of 3: 7, respectively. The final concentration of OmpA vaccine was set as 0.5 μg/μL and 1.0 μg/μL according to previous studies with some adjustments (24–27), and the concentration of inactivated vaccine was 1×10^8^ CFU/mL and 1×10^9^ CFU/mL (10, 24), respectively. Juvenile *M. amblycephala* (n = 450) were randomly divided into 5 groups (90 fish per group with 3 tanks), and the emulsified vaccines were intraperitoneally injected with 20 μL for each fish, then the pathological symptoms or deaths were continuously recorded for 2 weeks.

### 2.5 Fish rearing and vaccination

900 fish were divided into 5 groups (180 fish per group with 3 tanks): (I) control group (PBS instead of vaccines); (II) inactivated vaccine L group (1×10^8^ CFU/mL); (III) inactivated vaccine H group (1×10^9^ CFU/mL); (IV) OmpA vaccine L group (0.5 μg/μL); and (V) OmpA vaccine H group (1.0 μg/μL). As shown in **Table 1**, for the first immunization, the emulsions of mixed vaccines and adjuvant were intraperitoneally injected with 20 μL for each fish. Then, the booster immunization was conducted 2 weeks after the first vaccination, which was directly immunized with 20 μL PBS or vaccines without adjuvant.

**Table 1:** The immunization procedures and dosages.

Groups	Number of fish	First immunization (0 d) & booster immunization (14 d)			
		Vaccine	Vaccine concentration	Immunized dosage/μL	Adjuvant requirement
Control	180	PBS	0	20	First vaccination
Inactivated vaccine L	180	Inactivated vaccine	10^8^ CFU/mL	20	First vaccination
Inactivated vaccine H	180	Inactivated vaccine	10^9^ CFU/mL	20	First vaccination
OmpA vaccine L	180	OmpA subunit vaccine	0.5 μg/μL	20	First vaccination
OmpA vaccine H	180	OmpA subunit vaccine	1.0 μg/μL	20	First vaccination

The experimental fish were fed 4 times daily (8:00, 11:00, 14:00, and 17:00) to apparent satiation (approximately 3% of the body weight), and the water was renewed every day to maintain suitable water quality. The water temperature was maintained at 26-28°C with the dissolved oxygen greater than 6.0 mg/L, and the pH value was approximately 7.2, while ammonia, nitrogen, and nitrite were lower than 0.1 mg/L.

### 2.6 Bacterial challenge and sampling

The bacterial challenge assay was performed 2 weeks after the booster immunization as previously described (21), and experimental fish from each tank (60 fish/tank, 3 tanks/group) were assigned to 2 categories for calculating mortality (30 fish/tank) and sampling (30 fish/tank), respectively. The fish for sampling (body weight of 2.08 ± 0.25 g) were intraperitoneally injected with 20 μL of 1×10^7^ CFU/mL *A. hydrophila* (LD50 dose). Then, 3 individuals from each tank were randomly dissected after anesthetized with MS-222, and the blood, gill, hepatopancreas and intestines were collected at 0, 1, 3, 7, 14 and 30 d post infection (dpi). The fish for calculating mortality (body weight of 2.08 ± 0.25 g) were intraperitoneally injected with 20 μL of 5×10^7^ CFU/mL *A. hydrophila*, and the relative percent survival (RPS) was calculated using the formula: RPS = (1-% mortality in vaccinated fish/% mortality in control fish) × 100. Clinical signs and mortality were recorded everyday post infection for 14 d.

The blood was stored at 4°C overnight and centrifuged at 3000 rpm to isolate serum, which was used for Enzyme-linked immunosorbent assay (ELISA) assay and detection of enzymes activities, respectively. The hepatopancreas was homogenized and centrifuged at 4000 rpm for 10 min at 4°C for enzymes activities analysis. Immunohistochemical samples (gills, hepatopancreas and intestines) were fixed in 4% paraformaldehyde for 24 h at 4°C. Samples (gills, hepatopancreas and intestines) for RNA extraction were stored in sample protector for RNA (TaKaRa, Dalian, China) for 24 h at 4°C and then stored at -80°C until used.

### 2.7 Preparation and specificity verification of polyclonal antibodies

The recombinant IgM (AGR34023.1), IgD (AGR34025.1), IgZ (AGR34024.1) and CD8 (XP_048023282.1) protein were prepared in the same way as OmpA. After three immunizations with purified recombinant protein, rabbit antiserum against IgM, IgZ, IgD, and CD8 were obtained and purified using Protein A/G Agarose Kit (Beyotime, Shanghai, China). The total proteins of *M. amblycephala* tissues were extracted with RIPA lysate (Beyotime) and the concentration was determined with a BCA kit (Beyotime).

The specificity of prepared polyclonal antibodies was verified with purified recombinant proteins and total tissue proteins of *M. amblycephala* by western blotting (28). Specifically, protein samples were separated on a 12% SDS-PAGE gel and transferred onto polyvinylidene difluoride (PVDF) membranes using a Trans-Blot apparatus (BioRad, Berkeley, CA, USA). Non-specific reactivity was blocked with 5% (w/v) skim milk powder in TBS (150 mM NaCl, 20 mM Tris-base, pH 7.4). The PVDF membrane was incubated with polyclonal antibodies (1: 2000) and HRP-conjugated goat anti-rabbit IgG (H+L) (Beyotime; 1: 2000) for 1 h, respectively. Finally, a DAB Kit (Beyotime) was used for detection.

### 2.8 Enzyme-linked immunosorbent assay (ELISA)

The titer of serum immunoglobulin M (IgM) specific to *A. hydrophila* was detected by ELISA (29). In short, 96 well plates were coated with 200 μL of 5 μg/mL *A. hydrophila* ultrasonic fragments (same strain as used for inactivated vaccine preparation and challenge experiment) overnight at 4°C, and then blocked with 1% BSA at 22°C for 2 h, which were further incubated with *M. amblycephala* serum samples (1: 50 dilution) 96 well plates at 4°C for 12 h. Followed by incubating the 96 well plate with rabbit anti-*M. amblycephala* IgM specific antibody (1: 3000 dilution) at 22°C for 1 h, and incubating with goat anti-rabbit IgG-HRP secondary antibody (Beyotime; 1:2000 dilution) at 22°C for 1 h. After washing, the TMB substrate was added to incubate at 22°C for 5 min, then the termination solution was added to stop color reaction, and measured the absorbance at 450 nm. Internal positive and negative control samples were included. The relative OD values were calculated by the formula: Relative OD_450 nm_ = (OD_sample_ - OD_negative_) × (average of OD_positive_ of all plates)/OD_positive_.

### 2.9 Hematoxylin and eosin (H&E) staining

Three fixed tissues from each group were dehydrated with gradient ethanol, cleaned in xylene substitute, embedded in paraffin blocks, sectioned at 4 μm thickness, and stained with H&E (Sigma, St. Louis, Missouri, USA) (21). Three random fields of each slide were captured (400×) using a light microscope (Nikon, Tokyo, Japan). Histopathological change scores were obtained by evaluating the degree of tissue lesion. The score of 0 means asymptomatic, and the higher the score, the more serious the lesion.

### 2.10 Immunohistochemistry assay (IHC)

The expression and distribution of immune-related proteins (CD8, IgM, IgZ, and IgD) were detected by immunohistochemistry assay as previously described (28). Briefly, after dehydration, transparency and penetration, samples were embedded in paraffin. Sections (4 μm) were prepared, deparaffinized and rehydrated, then endogenous peroxidase was removed by immersion in 3% hydrogen peroxide. Sections were incubated with primary antibody (1: 2000) overnight at 4°C and then incubated with goat anti-rabbit IgG-HRP secondary antibody at 37°C for 30 min. The immunoreaction products were visualized using a DAB kit, and sections were counterstained with hematoxylin and embedded in glycerol and then positive cells were counted in three random fields using a light microscope (Nikon) with magnifications (×400 for hepatopancreas and intestine). The density of positive cells per mm^2^ was estimated as positive cells per mm^2^ (total of positive cells/total area counted).

### 2.11 Real-time quantitative reverse transcription polymerase chain reaction (qRT-PCR)

Total RNAs of collected samples were extracted using the RNA Easy Fast Tissue Kit (TIANGEN, Beijing, China) and the quality and concentration were determined by agarose gel electrophoresis and NanoDrop 2000 (Thermo Fisher Scientific, Wilmington, DE, USA), respectively. In addition, cDNA was synthesized using the PrimeScript^®^ RT reagent Kit with gDNA Eraser (TaKaRa) following the manufacturer’s protocol.

The expression patterns of *M. amblycephala* immune-related genes (*TNFα, iNOS, IL-1β, IL-6, IL-10, C3, SAA, TCR α, MHC I, MHC II, CD4, CD8, IgM, IgZ* and *IgD*) and *A. hydrophila 16S rRNA* gene were analyzed by qRT-PCR, as previously reported (28). Briefly, qRT-PCR was performed on the ABI StepOne Plus real-time PCR system (PerkinElmer Applied Biosystems, CA, USA) using the QuantiNova™ SYBR^®^ Green PCR Kit (TaKaRa). All the reactions were performed in triplicate with glyceraldehyde-3-phosphate dehydrogenase (*GAPDH)* as the reference gene, and the primers are listed in **Supplemental Table 1**. The relative expression levels of target genes were measured in terms of the threshold cycle (Ct) value using the 2^-ΔΔCt^ method (30). Gene expression levels of the control group were set as 1, and that of the vaccinated groups were presented as fold change. *A. hydrophila* abundance in different tissues of *M. amblycephala* from different groups was monitored by measuring *A. hydrophila 16S rRNA* transcripts levels, which was calculated according to the Ct values of qRT-PCR assay (29).

### 2.12 Analyses of antimicrobial enzymes activities

In this study, the activities of lysozyme (LZM), alkaline phosphatase (AKP), and acid phosphatase (ACP) in the serum and hepatopancreas were measured according to methods previously described (21). Briefly, according to the ratio of tissue weight (g) to phosphate buffer saline (PBS) volume (mL) of 1:9, hepatopancreas were homogenized. After centrifugation at 2500 rpm for 10 min, the supernatant was separated to determine the activities of antimicrobial enzymes. The activities of LZM, AKP and ACP were detected using the corresponding enzyme activity detection kit (Nanjing Jiancheng Bioengineering Institute, Nanjing, China).

### 2.13 Statistical analysis

In the present study, data were presented as mean ± standard error (SE). The statistical significance was assessed by one-way analysis of variance (ANOVA), and multiple comparisons were performed using the Tukey method of SPSS 25.0. The *P*-value of 0.05 was considered as statistically significant difference.

## 3 Results

### 3.1 Clinical symptoms and RPS

**Figure 1:**
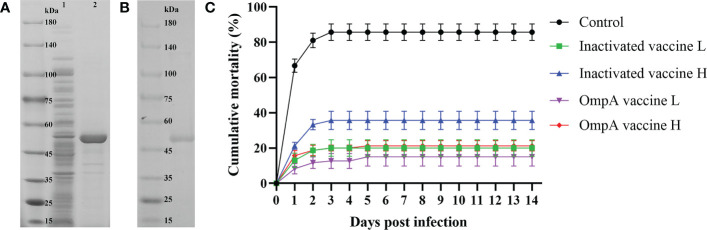
Preparation of recombinant OmpA protein and cumulative mortality. **(A)** SDS-PAGE analysis of the induced recombinant OmpA protein. Lane 1: total proteins of the uninduced *E coli* BL21 (DE3) competent cells that transformed with pET-32a-OmpA, Lane 2: total proteins of the induced *E coli* BL21 (DE3) competent cells that transformed with pET-32a-OmpA. **(B)** SDS-PAGE analysis of the purified recombinant OmpA protein. **(C)** Inactivated and OmpA subunit vaccines decreased the mortality of juvenile *M. amblycephala* post *A hydrophila* infection. Significant differences were found between the vaccinated groups and the control group (*P* < 0.05).

The recombinant OmpA protein was mainly expressed in the inclusion body with high purity (**Figures 1A, B**). The mortality rate in the control group was as high as 85.6% post *A. hydrophila* infection, while those of the inactivated vaccine L, inactivated vaccine H, OmpA vaccine L and OmpA vaccine H groups were 19.8%, 36%, 15% and 21%, respectively, which were significantly lower than that of the control group (**Figure 1C**). In addition, the RPS values of inactivated vaccine L, inactivated vaccine H, OmpA vaccine L and OmpA vaccine H groups were 76.9%, 58%, 82.3% and 75.5%, respectively. These results indicated that prepared vaccines showed excellent immunoprotective effects, especially OmpA vaccine L group.

Obvious clinical symptoms were observed in the control group at 1 dpi, including congestion of fish body, hemorrhage and swelling around anus and intestines, as well as severe hemorrhage and necrosis of gills. At 3 dpi, control fish presented obvious congestion of gills, and the intestines became thinned, brittle and were easy to break. In contrast, the symptoms of vaccinated fish mainly included slight congestion of gills at 1-3 dpi, and the intestines became thinned and brittle in the inactivated vaccine groups at 1 dpi (**Supplemental Figure 1**). After 7 dpi, all the survived fish gradually recovered and the clinical symptoms disappeared. The record of clinical symptoms revealed the immunoprotective effect of *A. hydrophila* vaccine.

### 3.2 Inactivated and OmpA subunit vaccines maintain the stability of histological structures of juvenile *M. amblycephala*

#### 3.2.1 Gill

**Figure 2:**
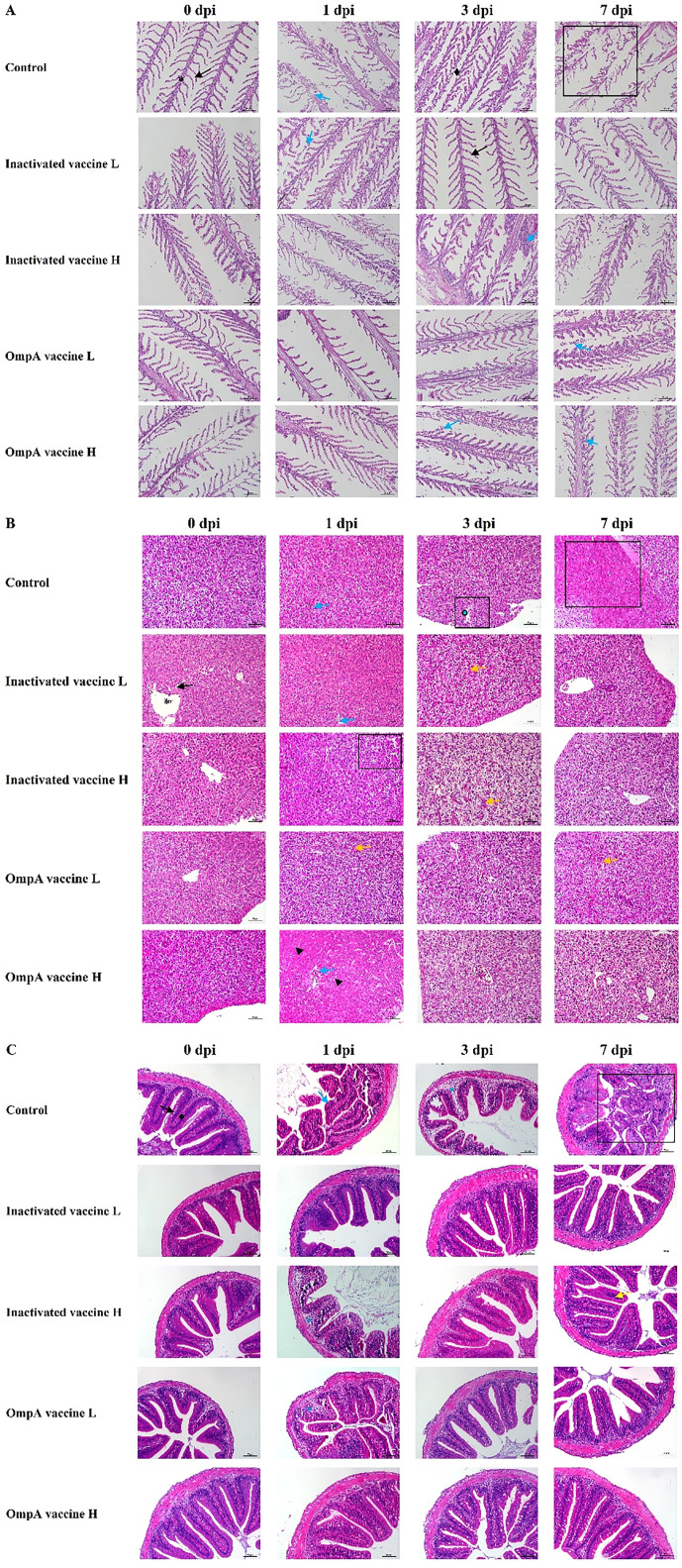
The histopathological changes post challenged by *A hydroophila.* Gills **(A)**, asterisk: gill filament; black arrow: gill lamellae; blue arrow: respiratory epithelium slough off; rhombuses: gill filament edema; rectangle: lamellae necrosis. Hepatopancreas **(B)**, asterisk: central vein; black arrow: hepatic sinusoid; blue arrow: congested central vein; yellow arrow: narrowed central vein, and intestines; triangle: degenerated hepatocytes; rectangle: focal necrosis. Intestine **(C)**, rhombuses: submucosa; black arrow: intestinal villi; asterisk: submucosa edema; blue arrow: intestinal villi necrosis; yellow arrow: inflammatory cells.

Histopathological changes of gill lamella congestion, necrosis, inflammatory cell infiltration and gill lamella hyperplasia were observed post infection. Three randomly selected fields (×400) were graded according to the histological changes (**Table 2**). Overall, hyperemia and inflammatory cell infiltration were found in gills lamellae of all fish at 1-7 dpi, while necrosis was commonly found in gills of control fish (**Figure 2A**). The gill lamella of all groups showed pathological characteristics at 1dpi. With the increase of infection time, the disease of gill lamella of immunized fish was alleviated and the tissue structure was improved, and the protective effect of OmpA vaccine was better than that of inactivated vaccine.

**Table 2:** Histopathological change scores in all study groups.

Tissues	0 dpi					1 dpi				
	control	inactivated vaccine L	inactivated vaccine H	OmpA vaccine L	OmpA vaccine H	control	inactivated vaccine L	inactivated vaccine H	OmpA vaccine L	OmpA vaccine H
H	0.00 ± 0	0.00 ± 0	0.00 ± 0	0.00 ± 0	0.00 ± 0	2.00 ± 0.58	5.67 ± 1.50	7.67 ± 0.33	8.33 ± 0.33	8.00 ± 0
G	3.67 ± 1.77	4.00 ± 1.73	2.67 ± 0.33	2.33 ± 0.33	1.67 ± 0.33	7.00 ± 0	4.67 ± 1.20	8.33 ± 2.67	3.00 ± 0	5.00 ± 2.30
I	0.00 ± 0	1.67 ± 1.67	0.00 ± 0	0.67 ± 0.67	0.00 ± 0	3.00 ± 1.53	3.33 ± 1.86	3.67 ± 1.20	2.00 ± 0	4.33 ± 1.86
	**3 dpi**					**7 dpi**				
H	8.00 ± 0.58	7.67 ± 0.33	7.33 ± 0.33	6.33 ± 0.33	7.33 ± 0.67	9.67 ± 1.20	8.00 ± 0	7.00 ± 0	7.33 ± 0.33	7.00 ± 0.58
G	3.67 ± 0.33	2.67 ± 0.88	3.67 ± 0.33	3.67 ± 0.33	4.33 ± 1.45	6.67 ± 2.67	5.00 ± 1.00	6.00 ± 1.00	5.00 ± 1.00	5.00 ± 1.00
I	1.67 ± 0.67	2.00 ± 0.58	1.33 ± 0.33	1.33 ± 0.33	1.00 ± 0	5.00 ± 3.06	1.00 ± 0	1.33 ± 0.33	1.00 ± 0	1.67 ± 0.33

Hepatopancreas (H), gills (G) and intestine (I).

#### 3.2.2 Hepatopancreas

Histopathological changes of hydropic degeneration, necrosis, sinusoidal congestion, inflammatory cell infiltration and eosinophilic staining were found in livers post infection. The severity of liver lesions was scored according to the histopathological changes as **Table 2**. Hydropic degeneration and focal necrosis were commonly observed in all livers at 1 dpi in both control and immunized groups. At 3 dpi, hydropic degeneration and rupture of hepatocytes leading to focal necrosis were evident in control livers, while the immunized livers displayed hepatocytes vacuolar degeneration and sinusoidal congestion. At 7 dpi, hepatocytes of control livers presented diffuse necrosis, whereas only focal necrosis and sinusoidal congestion were observed (**Figure 2B**).

#### 3.2.3 Intestine

After bacterial infection, the major histopathological changes of intestine were intestinal villus necrosis (1 dpi) and submucosal edema (3 dpi) in control group, while the immunized intestine only displayed submucosa edema at 1 dpi (except for a small inflammatory cell infiltration at 7 dpi in inactivated vaccine H group) (**Figure 2C**). The severity was scored according to the intestinal lesions as **Table 2**. The intestinal lesions of all groups were relatively high at 1dpi, which were significantly alleviated in the next few days in all immunized groups.

### 3.3 Inactivated and OmpA subunit vaccines increase the production of IgM specific to *A. hydrophila*

**Figure 3:**
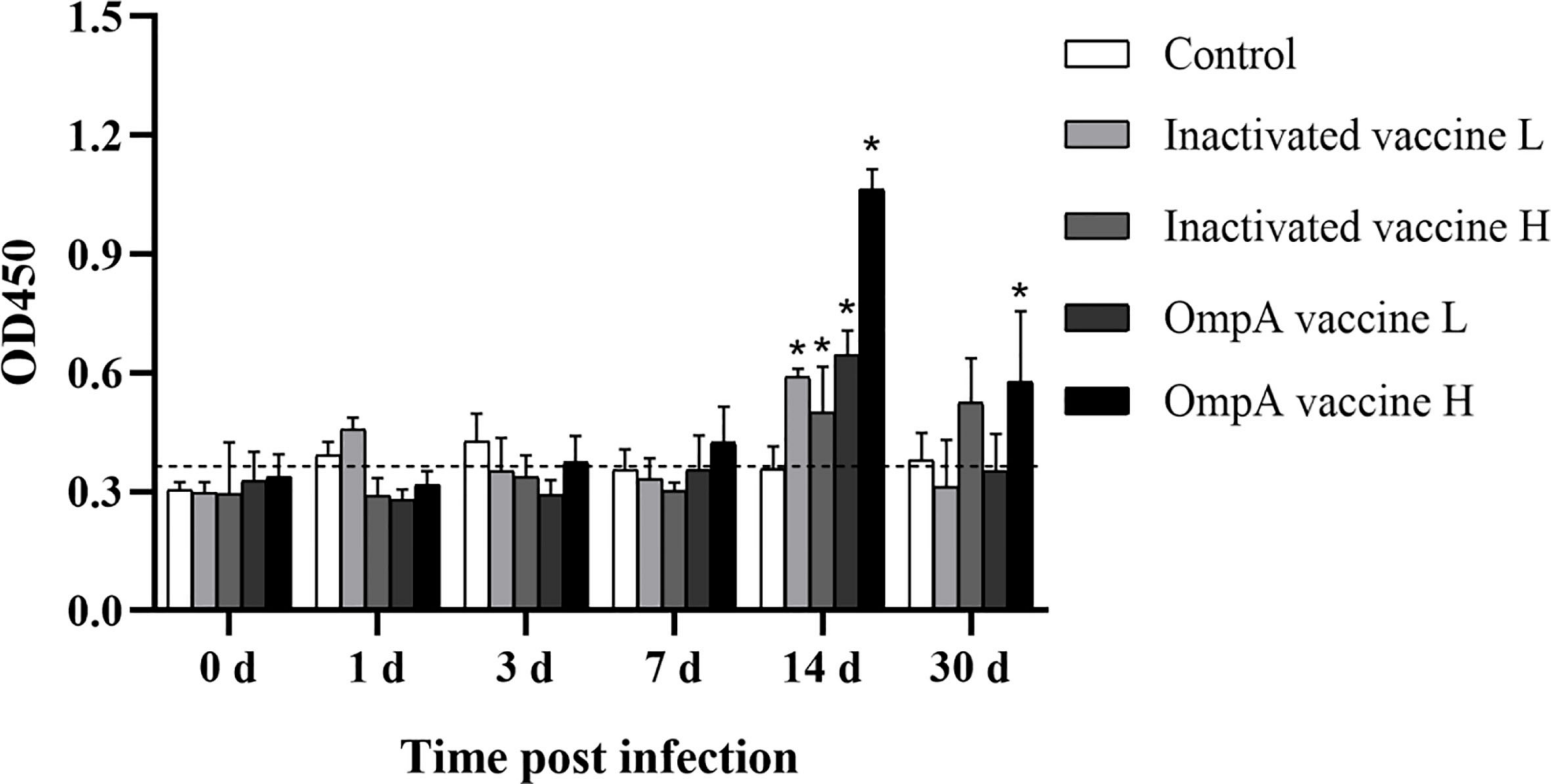
The specific IgM levels to *A. hydrophila* detected by ELISA. Asterisk (*) indicated significant difference between the vaccinated groups and control group (*P* < 0.05). The vertical cut-off line represented the control serum + 3×standard deviation.

The specificity of prepared anti-IgM antibody and no cross reaction between IgD, IgZ and IgM was verified by western blotting (**Supplemental Figures 2A, E**). No significant difference in IgM levels between control and vaccinated groups before and at 7 dpi, with just slight fluctuations. At 14 dpi, the specific IgM levels in the vaccinated groups were up-regulated to the peak and higher than that of the control group, especially these of the two OmpA subunit vaccine groups were more significant (**Figure 3**), which indicated that vaccination increased the production of specific antibodies and enhanced host resistance to *A. hydrophila* infection.

### 3.4 Inactivated and OmpA subunit vaccines decrease the abundance of *A. hydrophila* in various tissues

**Figure 4:**
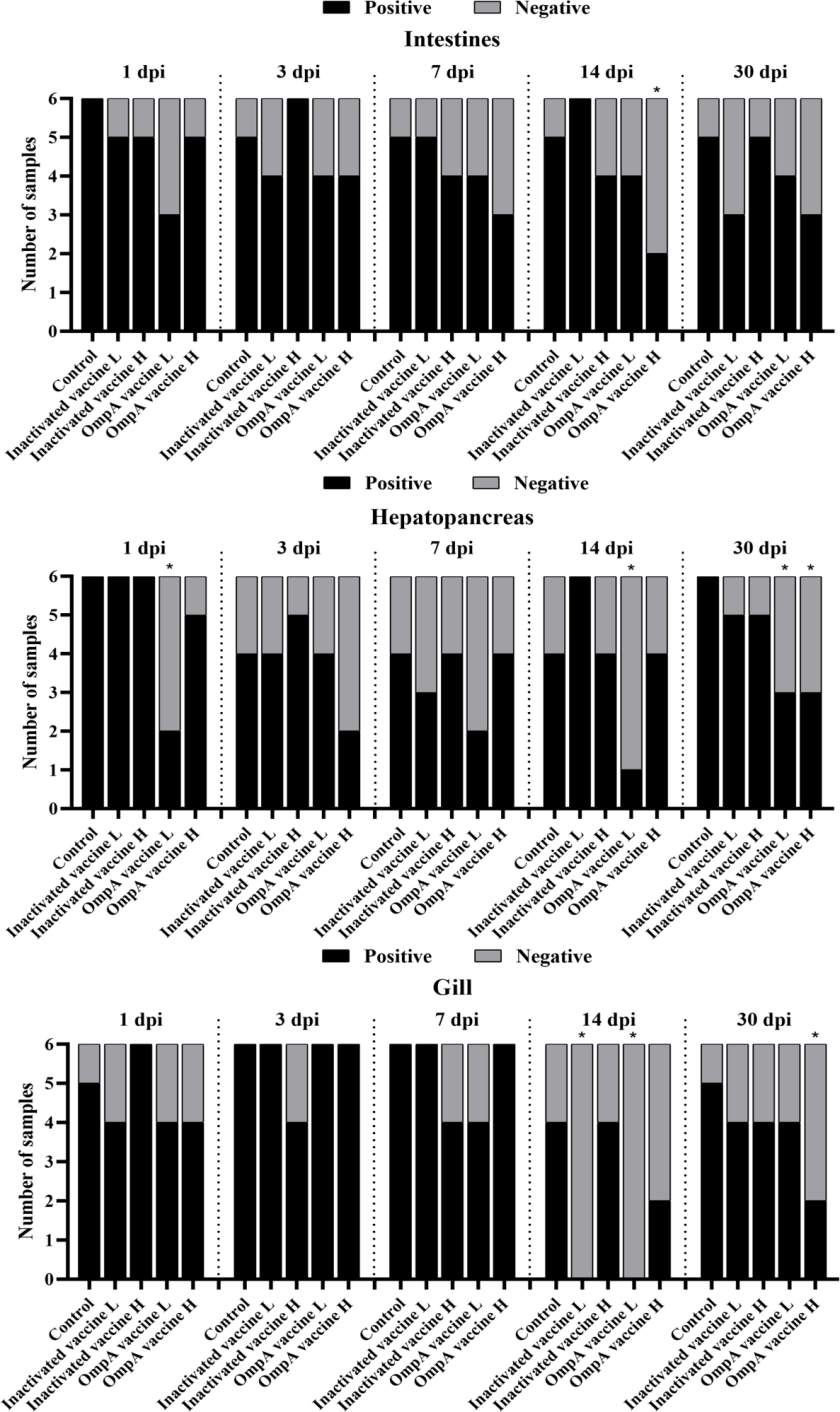
Infection of *M. amblycephala* in experimental groups expressed as percentage of fish carrying *A. hydrophila* in different organs following challenge quantified by RT-qPCR with *16S rRNA*. *: Significant difference compared to the control group at each time point (*P* < 0.05).

The *16S rRNA* transcripts levels of *A. hydrophila* in different tissues were detected to compare the bacterial abundance in different groups at various time points post infection. In the intestines, *16S rRNA* transcripts of most vaccinated groups were lower than that of the control group, especially these of the OmpA vaccine groups at all time points. The OmpA vaccine groups also showed lower levels of *16S rRNA* transcripts in the hepatopancreas and gills. However, the up-regulated levels of *16S rRNA* transcripts were observed in the gills at 3 and 7 dpi of most groups, which should be related to the method of intraperitoneal injection. Overall, these results revealed that vaccines immunization (especially OmpA vaccines) decreased the *A. hydrophila* abundance in the host, thereby protecting juvenile *M. amblycephala* against infection (**Figure 4**).

### 3.5 Inactivated and OmpA subunit vaccines increase immune cell quantity

**Figure 5:**
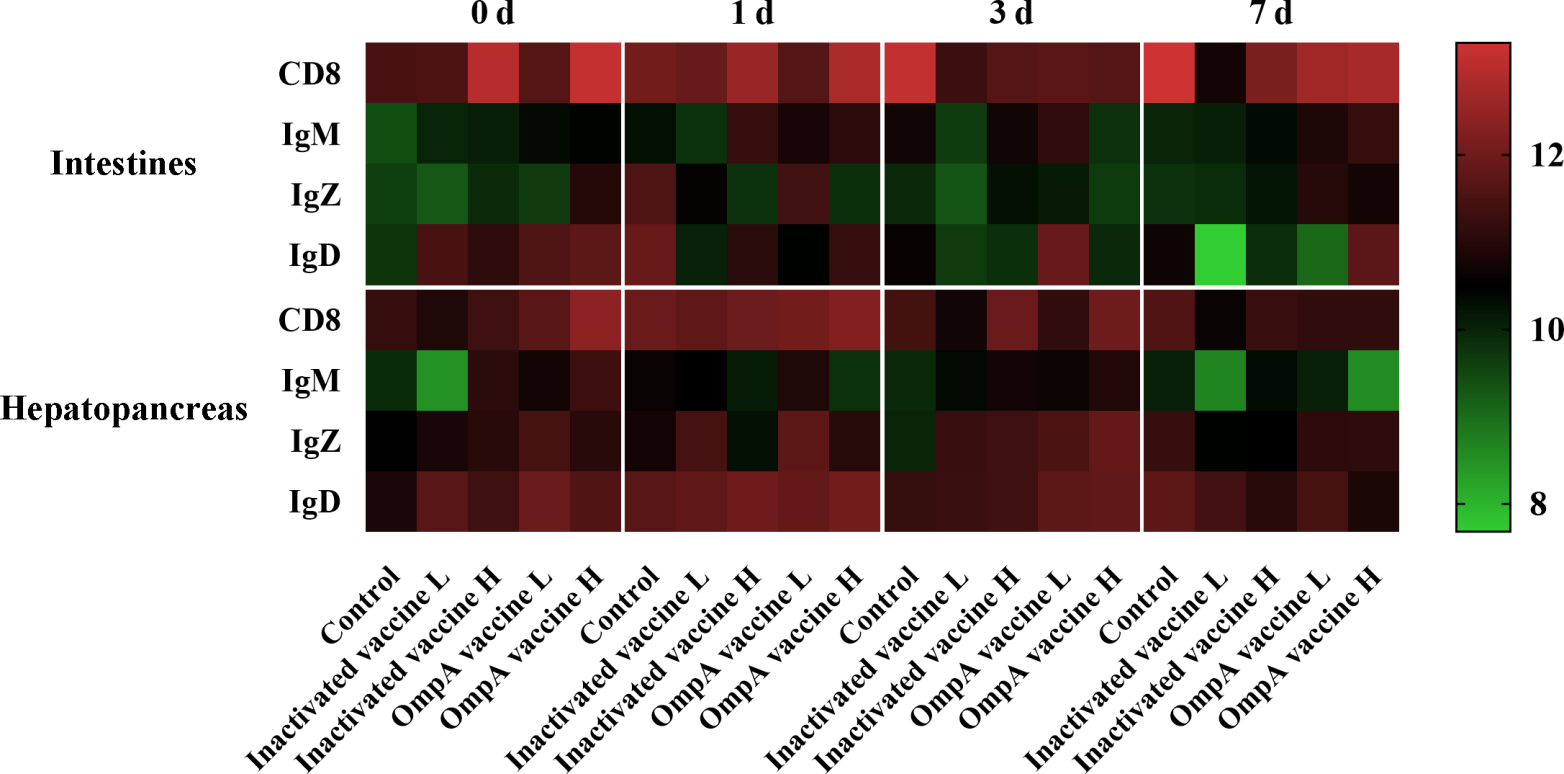
Heatmap illustrates results from IHC assay for the number of immune positive cells that affected by vaccination in the hepatopancreas and intestines at different time points post infection. Color value: log2 (fold change).

The specificity of all prepared antibodies was validated by western blotting which showed excellent specificity (**Supplemental Figure 2**). Four immune-related proteins (CD8, IgM, IgZ and IgD) showed strong positive immunoreactive signals in both tissues (**Table 3**). In general, the number of immune positive cells was much more abundant in the hepatopancreas than in the intestines (except CD8^+^ cells), which also maintained abundant levels post infection, while that in the intestines just showed partial up-regulation post infection (**Figure 5**).

**Table 3:** IHC assay revealed the effects of vaccination on the number of immune positive cells in the hepatopancreas and intestines at different time points post infection.

Name of group	CD8		IgD		IgM		IgZ		Day (dpi)
	I	H	I	H	I	H	I	H	
Control	**2838 ± 209**	**2432 ± 478**	**870 ± 61**	**2072 ± 762**	**699 ± 90**	**978 ± 167**	**782 ± 31**	**1597 ± 523**	**0**
Inactivated vaccine L	**2960 ± 242**	**2036 ± 462**	**2860 ± 379**	**3298 ± 242**	**1026 ± 128**	**362 ± 10**	**628 ± 24**	**2162 ± 739**
Inactivated vaccine H	**8026 ± 406**	**2594 ± 228**	**2234 ± 240**	**2582 ± 140**	**1124 ± 251**	**2361 ± 681**	**969 ± 122**	**2102 ± 116**
OmpA vaccine L	**3141 ± 107**	**3404 ± 636**	**3091 ± 503**	**3955 ± 695**	**1313 ± 134**	**1721 ± 119**	**833 ± 113**	**2744 ± 18**
OmpA vaccine H	**9050 ± 268**	**5420 ± 714**	**3399 ± 185**	**3070 ± 151**	**1391 ± 116**	**2565 ± 170**	**2036 ± 163**	**2100 ± 94**
Control	**4196 ± 282**	**4077 ± 957**	**4029 ± 944**	**3279 ± 280**	**1321 ± 322**	**1564 ± 164**	**3017 ± 229**	**1901 ± 618**	**1**
Inactivated vaccine L	**3774 ± 143**	**3492 ± 208**	**1061 ± 145**	**3505 ± 280**	**898 ± 99**	**1440 ± 137**	**1497 ± 101**	**2755 ± 167**
Inactivated vaccine H	**6115 ± 609**	**3975 ± 400**	**2123 ± 190**	**4179 ± 680**	**2375 ± 279**	**1092 ± 25**	**897 ± 87**	**1271 ± 268**
OmpA vaccine L	**3228 ± 590**	**4211 ± 157**	**1403 ± 97**	**3668 ± 66**	**1858 ± 339**	**1893 ± 10**	**2670 ± 129**	**3480 ± 397**
OmpA vaccine H	**7076 ± 147**	**4882 ± 415**	**2378 ± 268**	**4256 ± 657**	**2154 ± 197**	**896 ± 88**	**920 ± 57**	**2109 ± 340**
Control	**8899 ± 257**	**2961 ± 778**	**1541 ± 134**	**2512 ± 612**	**1709 ± 252**	**1012 ± 207**	**968 ± 30**	**1060 ± 194**	**3**
Inactivated vaccine L	**2491 ± 187**	**1686 ± 20**	**828 ± 56**	**2481 ± 203**	**816 ± 71**	**1329 ± 170**	**649 ± 78**	**2408 ± 17**
Inactivated vaccine H	**3192 ± 467**	**3889 ± 351**	**924 ± 25**	**2725 ± 589**	**1726 ± 252**	**1700 ± 68**	**1240 ± 141**	**2657 ± 458**
OmpA vaccine L	**3328 ± 168**	**2238 ± 133**	**3842 ± 416**	**3442 ± 317**	**2248 ± 34**	**1634 ± 184**	**1198 ± 294**	**2931 ± 249**
OmpA vaccine H	**3127 ± 122**	**4015 ± 346**	**1014 ± 217**	**3594 ± 449**	**895 ± 61**	**1947 ± 203**	**813 ± 20**	**3694 ± 343**
Control	**9973 ± 586**	**3331 ± 888**	**1676 ± 248**	**3485 ± 251**	**1028 ± 68**	**1067 ± 153**	**932 ± 193**	**2391 ± 192**	**7**
Inactivated vaccine L	**1827 ± 458**	**1669 ± 388**	**226 ± 62**	**2801 ± 550**	**1100 ± 205**	**427 ± 95**	**945 ± 107**	**1423 ± 206**
Inactivated vaccine H	**4560 ± 403**	**2457 ± 287**	**938 ± 68**	**2166 ± 459**	**1298 ± 148**	**1275 ± 103**	**1167 ± 84**	**1588 ± 462**
OmpA vaccine L	**6471 ± 257**	**2295 ± 369**	**532 ± 61**	**2722 ± 57**	**1952 ± 392**	**1110 ± 246**	**2078 ± 281**	**2400 ± 707**
OmpA vaccine H	**7008 ± 485**	**2320 ± 393**	**3380 ± 203**	**2048 ± 574**	**2365 ± 189**	**382 ± 37**	**1739 ± 226**	**2352 ± 519**

The number of positive cells was calculated in an area of 1 mm^2^. Counting of each cell type was performed on 3 fish/group at 0, 1, 3 and 7 dpi. Red shading indicates a significant increase in positive cells, and green shading indicates a significant decrease in positive cells *(P < 0.05)*. Intestine (I) and hepatopancreas (H).

#### 3.5.1 CD8 α^+^ cells

The CD8 α^+^ cells were more abundant in vaccinated groups before infection (**Supplemental Figures 3A, 4A, 5A**) and significantly increased in all groups post infection, especially the control group that maintained at relatively high level. In general, whether challenged or not, the number and trend of CD8 α^+^ cells were consistent in vaccinated groups, which was more abundant in the high-dose vaccines groups.

#### 3.5.2 IgD^+^ cells

The IgD^+^ cells were also more abundant in vaccinated groups before bacterial infection in the hepatopancreas and intestines (**Supplemental Figures 3B**, **4B**, **5B**), which also maintained abundant levels in the hepatopancreas of all groups post infection. In the intestines, the IgD^+^ cells of control group only increased at 1 dpi, while it is more abundant in the OmpA vaccine immunized groups at 3 or 7 dpi.

#### 3.5.3 IgM^+^ cells

In general, the abundance of IgM^+^ cells was much lower in both tissues at all time points compared to other immune cells (**Supplemental Figures 3C**, **4C**, **5C**). In addition, the number of IgM^+^ cells was more abundant in the vaccinated groups compared with control group before infection and was not affected in control group post infection. Moreover, the abundance of IgM^+^ cells was a bit up-regulated in the intestines of vaccinated groups post infection, while these in the hepatopancreas decreased.

#### 3.5.4 IgZ^+^ cells

In the hepatopancreas, the number of IgZ^+^ cells was higher in immunized groups before infection, which also maintained abundant levels post infection, and the up-regulation in the control group was only observed at 7 dpi (**Supplemental Figures 3D**, **4D**, **5D**). Additionally, the abundance of IgZ^+^ cells was generally lower in the intestines than that in the hepatopancreas, with only partial up-regulation found in the control and OmpA vaccine L groups at 1 dpi.

### 3.6 Inactivated and OmpA subunit vaccines promote the expression of immune related genes

**Figure 6:**
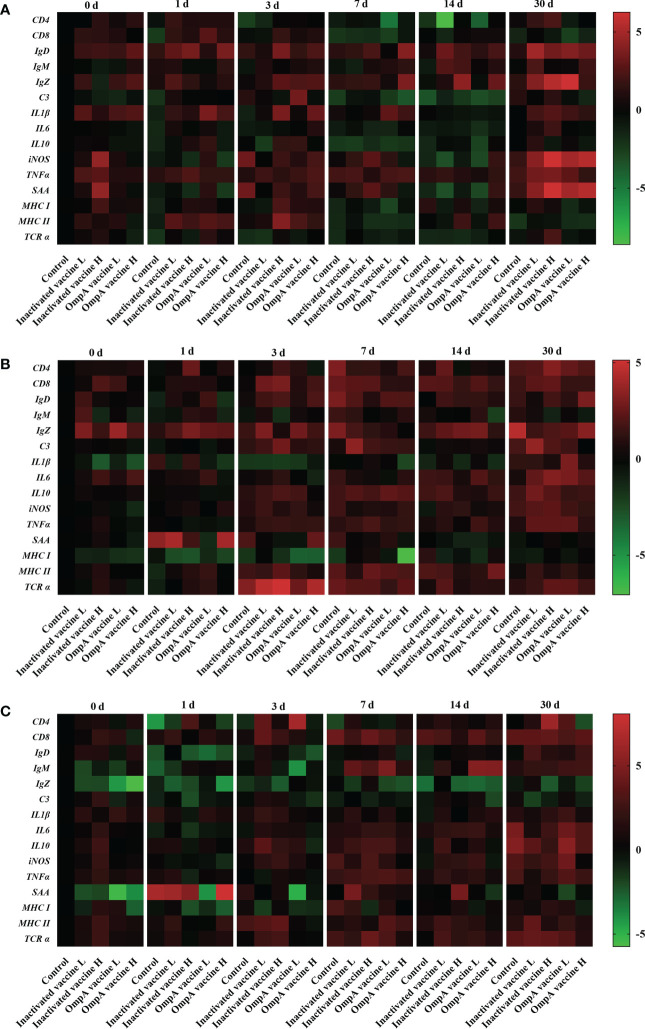
Heatmaps illustrate results from qRT-PCR for the expression of immune related genes in the intestines **(A)**, gills **(B)** and hepatopancreas **(C)** of juvenile *M. amblycephala*. Color value: log2 (fold change).

In the intestines, the expression of adaptive immune-related genes (*C3, TCR α, MHC I, MHC II, CD4, CD8, IgM, IgZ* and *IgD*) was higher in immunized groups after 2 times vaccination, and most of them were also significantly up-regulated in the vaccinated groups post infection, except for *CD4* and *TCR α* (**Figure 6A**; **Supplemental Figure 6A**). In addition, proinflammatory cytokines (*TNFα, iNOS, IL-1β, IL-6* and *SAA*) showed similar expression patterns to adaptive immune-related genes, especially the significant up-regulation of acute phase protein *SAA*, while the expression of anti-inflammatory cytokine *IL-10* was mainly decreased post infection. These results indicated that vaccine immunization enhanced host innate and adaptive immunity in the intestines, especially the high dose vaccine groups.

In the gills, the expression of most adaptive immune-related genes was also higher in immunized groups after vaccination and post infection, except for *MHC I* (**Figure 6B**; **Supplemental Figure 6B**). The expression of most proinflammatory cytokines was relatively low after vaccination, but up-regulated significantly at 3 and 7 dpi (*SAA* up-regulated significantly at 1 dpi). These results also revealed that vaccine immunization strengthened host innate and adaptive immunity in the gills.

In the hepatopancreas, similar expression patterns were found in adaptive and innate immune-related genes with low expression levels before 1 dpi, which started up-regulated from 3 dpi to 30 dpi (except for *IgZ* and *MHC I*). In addition, the vaccinated groups showed higher expression levels than those of the control group at most time points, indicating that vaccine immunization also enhanced host immunity (**Figure 6C**; **Supplemental Figure 6C**).

### 3.7 Inactivated and OmpA subunit vaccines enhance the activities of antimicrobial enzymes

**Figure 7:**
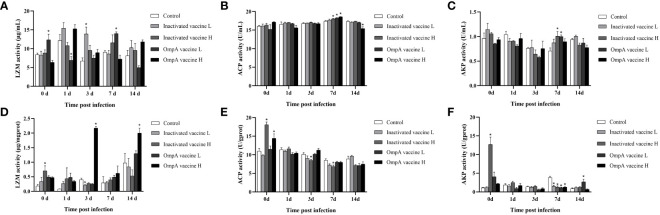
Effects of vaccination on the activities of serumal and hepatic antimicrobial enzymes of juvenile *M. amblycephala* upon infection. **(A–C)** represented the activities of LZM, ACP and AKP in the serum, respectively. **(D–F)** represented the activities of LZM, ACP and AKP in the hepatopancreas, respectively. Asterisk (*) represented a significant difference between the vaccinated groups and control group (*P* < 0.05).

The activities of LZM, ACP and AKP in the hepatopancreas and serum of juvenile *M. amblycephala* post infection were detected to evaluate effects of vaccination on host antibacterial ability. In the serum, the LZM activities of most groups reached the peak levels at 1 dpi, and immunized groups showed much higher activities compared with control group (*P* < 0.05). In the hepatopancreas, all vaccinated groups showed much higher LZM activities at 0, 1 and 7 dpi. Although LZM activity of the control group increased significantly at 3 and 14 dpi, LZM activity of the OmpA vaccine groups was dramatically higher (*P* < 0.05). In general, the LZM activities of all groups were up-regulated post infection in hepatopancreas and serum (**Figures 7A, D**), especially vaccinated groups, indicating that vaccine immunization enhanced the host antibacterial ability.

The activities of ACP showed different levels and trends in the hepatopancreas and serum, which maintained high and stable levels in the serum upon infection, and the control and vaccinated groups showed no significant difference with just a partial increase at 7 dpi (**Figures 7B, E**). In contrast, the hepatic ACP activities of inactivated vaccine H and OmpA vaccine H groups were higher than that of the control group at 0 dpi, which gradually decreased post infection. Thus, these results indicated that the bactericidal effect of serum was more excellent than that of hepatopancreas, and vaccination significantly enhanced the bactericidal effect of serum post infection, but not that of hepatopancreas.

The activities of AKP were fluctuant within limits in both serum and hepatopancreas, except for the extremely significant up-regulation of vaccinated groups in the hepatopancreas at 0 dpi, but the regulation of vaccination was also inconsistent at various time points post infection, indicating that the variable effects of vaccination on inflammatory response upon infection. In a word, vaccines immunization (especially OmpA vaccines) enhanced the antibacterial ability of juvenile *M. amblycephala* against *A. hydrophila* infection (**Figures 7C, F**).

## 4 Discussion

Vaccine immunization is an important method for disease prevention and control in aquaculture, and more than 140 kinds of aquatic vaccines have been approved for marketing in the world (31). The inactivated *A. hydrophila* vaccines always provide excellent immune protective effects by inducing immune responses of aquatic animals, which thereby were widely studied and applied (32). However, due to wide range of sources and serotypes of *A. hydrophila*, which limit the broad-spectrum immune protective effects and application scopes of inactivated *A. hydrophila* vaccines. Studies have shown that the outer membrane proteins of *A. hydrophila* have high immunogenicity, which can stimulate the humoral and cellular immunity of fish. In addition, they are highly conserved among different strains, thus are ideal antigens for preparing *A. hydrophila* vaccines with broad-spectrum immune protection (14, 15).

The immunized recombinant Aha1 and OmpW proteins show significant protective effects on the carp upon *A. hydrophila* infection with the production of specific antibodies (14). Similarly, oral immunization of rOmpW protein also shows protective immunity in a dose-dependent manner (33). Indian carp immunized with OmpC subunit vaccine also achieves immune protective effect, and detectable cross-reacts of rOmpC antiserum with different *Aeromonas* lysates (34). In addition, other studies have evaluated the high immunogenicity of *A. hydrophila* OMPs, including Omp38 (24), OmpA1 (26), OmpF (35), Aha1 (36). In this study, we evaluated the relative survival rate of the prepared *A. hydrophila* inactivated vaccine and OmpA subunit vaccine upon bacterial infection, and both vaccines exhibited excellent immune protection to the juvenile *M. amblycephala*, among which the OmpA subunit vaccine showed better immune protective effect than that of the inactivated vaccine.

The protective effect of *A. hydrophila* inactivated vaccine and OmpA subunit vaccine should be correlated with the increased production of specific antibody, the decreased abundance of pathogen in the host, the enhanced expression and distribution of immune genes, improved histological structures and activities of antibacterial enzymes in *M. amblycephala*. In this study, the vaccines immunization could effectively reduce the pathological characteristics and damage of tissues caused by *A. hydrophila* infection and maintain the integrity of tissues, thereby protecting the host from inflammatory damage, which has also been observed in *Cirrhinus mrigala* and *C. idella* (37). Serum immunoglobulin IgM is the main antibody produced fish post infection or pathogenic antigen stimulation, which has the functions of dissolving bacteria, activating complements, immune conditioning and agglutination, and also plays a role in clearing pathogens at the early stage of infection. In this study, the up-regulated production of specific antibody against *A. hydrophila* in the vaccinated groups (especially OmpA vaccines) could enhance the host adaptive immunity, thereby decreasing the cumulative mortality post bacterial infection and exhibiting excellent immune protective effects, which is consistent with the studies in *I. punctatus* and *P. mesopotamicus* (10, 26). Dietary probiotics as an immunomodulator can also increase the level of IgM in the serum of *Mycteroperca rosacea* (38). Similarly, when the content of arginine in feed increases, the production of IgM is accelerated, thus the disease resistance of experimental fish is improved (39).

Previous study has revealed that the mean bacterial concentrations in immune tissues were significantly lower in *I. punctatus* vaccinated with the fimbrial or OmpA1 than in the non-vaccinated control group (26, 40). Similarly, the present study found the abundance of *A. hydrophila* in the tissues of juvenile *M. amblycephala* was affected by vaccination, which was significantly down-regulated in the vaccines immunized groups, especially that of the OmpA vaccine groups, indicating that vaccines immunization could reduce the proliferation of *A. hydrophila* in the host and thereby protecting host from inflammatory damage and death.

The specific immune response is usually studied to determine the effect of vaccine immunization on bacteria-infected organisms, but some studies have shown that vaccination also has a positive effect on the non-specific immune response, which is also an important defense mechanism to protect fish from bacterial infection. Antimicrobial enzymes such as LZM, ACP and AKP are common indicators for evaluating host nonspecific immunity (41). LZM has high bactericidal or hemolytic activity against pathogens, and ACP and AKP may contribute to promoting immunogenic responses (42–44), which thereby play vital roles in host defense system. In the present study, the levels of LZM, ACP and AKP in vaccinated groups were up-regulated, which was similar to the results in *C. carpio* with *A. hydrophila* Aha1 vaccine immunization and other previous studies, indicating that both OmpA subunit vaccine and inactivated vaccine can improve host non-specific immunity and enhance their ability to resist infection (10, 36).

It has been proved that hepatopancreas, intestines and gills are important immune tissues in fish, which participate in host immunity, thereby immune related genes in these tissues play important roles in the immune defense responses post bacterial infection. The present study revealed that the number of CD8, IgD, IgZ, and IgM positive cells in the intestine and hepatopancreas increased post infection, especially that of the vaccinated groups, indicating that vaccines immunization activated host adaptive immune system and thus enhanced bacterial clearance ability. Consistently, the present study found that the expression of adaptive immune-related genes (*C3, TCR α, MHC I, MHC II, CD4, CD8, IgM, IgZ* and *IgD*) were higher in the vaccines immunized groups after 2 times vaccination, and most of them were also significantly up-regulated in the vaccinated groups post infection. Similar expression patterns of *MHC I* and *MHC II* genes were also observed in *Pseudosciaena crocea* (45), *Salmo salar* (46) and *M. amblycephala* (47).

CD8^+^ and CD4^+^ T cells use the T cell receptors (TCRs) to recognize complexes of pathogen-specific peptides antigens that are presented by major histocompatibility (MHC) class I and class II molecules on the surface of infected cells, respectively (48). Therefore, *CD4* and *CD8* genes are up-regulated in the immune-related tissues of the juvenile *M. amblycephala* post infection, which might interact with MHC class II or I molecule and play an important role in the immune response. In previous study, CD8^+^ T cells play a role in the adaptive immunity of *C. auratus* against parasites infection, especially in the kidney and gills (49). It was also observed that the leukocytes of fish infected with virus showed high level of cytotoxicity and abundantly expressed *CD8 α* and *TCR β*, exerting specific immune functions (50). Immunoglobulins (Igs) are important molecules in fish adaptive immune system with high specificity, which were up-regulated when *M. amblycephala* infected by *A. hydrophila* (51–53). Consistent with this study, *C. idella IgM* gene expression was also induced after vaccination of single-walled carbon nanotubes (SWCNTs) coated *A. hydrophila* aerA subunit vaccine (25).

In the present study, expression of inflammatory cytokines (*IL-1 β, IL-6, IL-10, TNF α and iNOS*) and acute phase protein *SAA* were up-regulated post infection, especially that of the vaccines immunized groups, indicating that vaccination promoted host innate immunity of *M. amblycephala*. Similarly, the recombinant *A. hydrophila* OmpF vaccine immunized *Labeo rohita* also showed induced the expression of the immune-related genes such as *IL-1 β* and *TNF α* in the head kidney tissues, when compared to the control group at different time points post vaccination (35). These studies revealed that *A. hydrophila* vaccines immunization could enhance the innate immunity of fish, thus improving host resistance to bacterial infection.

## 5 Conclusions

Herein, we comparatively evaluated the immunogenicity and protective effects of *A. hydrophila* inactivated vaccine and OmpA subunit vaccine in *M. amblycephala* for the first time, and presented more comprehensive evaluation index. This study revealed that both vaccines (especially OmpA subunit vaccine) exhibited excellent protective effects on juvenile *M. amblycephala*, which should attribute to the increased production of specific antibody, and the decreased abundance of *A. hydrophila* in the tissues, and the enhanced expression of immune-related genes, and the improved histological structures and antibacterial enzymes activities. Therefore, both *A. hydrophila* inactivated vaccine and OmpA subunit vaccine are efficient vaccines, while OmpA subunit vaccine is a more ideal candidate vaccine against *A. hydrophila*.

## Data availability statement

The original contributions presented in the study are included in the article/**Supplementary Material**. Further inquiries can be directed to the corresponding author.

## Ethics statement

The animal study was reviewed and approved by the Animal Care and Use Committee of Jiangsu Ocean University.

## Author contributions

Conceptualization, ZD and XZ. Methodology, HL. Software, HpL and ZX. Validation, JX. Formal analysis, XW. Investigation, MZ, TZ, and HJC. Resources, XC. Data curation, YH. Writing—original draft preparation, MZ. Writing—review and editing, ZD and XZ. Visualization, MZ and YL. Supervision, ZD and XZ. Project administration, XZ. Funding acquisition, ZD, XZ, and HLC. All authors contributed to the article and approved the submitted version.

## References

[1] Fang HM Ge R Sin YM. Cloning, characterisation and expression of *Aeromonas hydrophila* major adhesin. Fish Shellfish Immunol (2004) 16(5):645–58. doi: 10.1016/j.fsi.2003.10.003 15110338

[2] Su H Su J. Cyprinid viral diseases and vaccine development. Fish Shellfish Immunol (2018) 83:84–95. doi: 10.1016/j.fsi.2018.09.003 30195914 PMC7118463

[3] Buckley JT Howard SP. The cytotoxic enterotoxin of *Aeromonas hydrophila* is aerolysin. Infect Immun (1999) 67(1):466–7. doi: 10.1128/IAI.67.1.466-467.1999 PMC96339 9925450

[4] Shoemaker CA Mohammed HH Bader TJ Peatman E Beck BH. Immersion vaccination with an inactivated virulent *Aeromonas hydrophila* bacterin protects hybrid catfish (*Ictalurus punctatus* × *Ictalurus furcatus*) from motile *Aeromonas* septicemia. Fish Shellfish Immunol (2018) 82:239–42. doi: 10.1016/j.fsi.2018.08.040 30130658

[5] Khushiramani R Girisha SK Karunasagar I Karunasagar I. Cloning and expression of an outer membrane protein ompTS of *Aeromonas hydrophila* and study of immunogenicity in fish. Protein Expr Purif (2007) 51(2):303–7. doi: 10.1016/j.pep.2006.07.021 16959494

[6] Zeng H Xie M Ding C Ma J Xu D Wang X. Attenuated *Listeria monocytogenes* as a vaccine vector for the delivery of OMPW, the outer membrane protein of *Aeromonas hydrophila* . Front Microbiol (2020) 11:70. doi: 10.3389/fmicb.2020.00070 32153514 PMC7047129

[7] Kurath G. Biotechnology and DNA vaccines for aquatic animals. Rev Sci Tech (2008) 27(1):175–96. doi: 10.20506/rst.27.1.1793 18666487

[8] Chandran MR Aruna BV Logambal SM Michael RD. Immunisation of Indian major carps against *Aeromonas hydrophila* by intraperitoneal injection. Fish Shellfish Immunol (2002) 13(1):1–9. doi: 10.1006/fsim.2001.0374 12201649

[9] Bharadwaj A Abraham TJ Joardar SN. Immune effector activities in challenged rohu, *Labeo rohita* after vaccinating with *Aeromonas* bacterin. Aquaculture (2013) 392:16–22. doi: 10.1016/j.aquaculture.2013.01.016

[10] Vaz Farias TH Arijo S Medina A Pala G da Rosa Prado EJ Montassier HJ. Immune responses induced by inactivated vaccine against *Aeromonas hydrophila* in pacu, *Piaractus mesopotamicus* . Fish Shellfish Immunol (2020) 101:186–91. doi: 10.1016/j.fsi.2020.03.059 32247044

[11] Liu YJ Bi Z. Potential use of a transposon Tn916-generated mutant of *Aeromonas hydrophila* J-1 defective in some exoproducts as a live attenuated vaccine. Prev Vet Med (2007) 78(1):79–84. doi: 10.1016/j.prevetmed.2006.09.004 17079040

[12] Han B Xu K Liu Z Ge W Shao S Li P. Oral yeast-based DNA vaccine confers effective protection from *Aeromonas hydrophila* infection on *Carassius auratus* . Fish Shellfish Immunol (2019) 84:948–54. doi: 10.1016/j.fsi.2018.10.065 30445667

[13] Nayak SK. Current status of *Aeromonas hydrophila* vaccine development in fish: An Indian perspective. Fish Shellfish Immunol (2020) 100:283–99. doi: 10.1016/j.fsi.2020.01.064 32088285

[14] Maiti B Shetty M Shekar M Karunasagar I Karunasagar I. Evaluation of two outer membrane proteins, Aha1 and OmpW of aeromonas hydrophila as vaccine candidate for common carp. Vet Immunol Immunopathol (2012) 149(3-4):298–301. doi: 10.1016/j.vetimm.2012.07.013 22917476

[15] Maji S Mali P Joardar SN. Immunoreactive antigens of the outer membrane protein of *Aeromonas hydrophila*, isolated from goldfish, *Carassius auratus* (Linn.). Fish Shellfish Immunol (2006) 20(4):462–73. doi: 10.1016/j.fsi.2005.06.003 16243540

[16] Jiang W Liu Y Lu C. Expression and immunogenicity analysis of the fusion protein OmpA from *Aeromonas hydrophila* strain J-1. J Fish Sci China (2008) 15(2):301–6. doi: 10.3321/j.issn:1005-8737.2008.02.014

[17] Zheng Z Zheng S Delbert MG Wang K. Prokaryotic expression of outer membrane protein a (ompA) gene of *Aeromonas hydrophila* and its immunoprotection. Freshw Fish (2015) 45(5):3–10. doi: 10.3969/j.issn.1000-6907.2015.05.001

[18] Ma J Bruce TJ Sudheesh PS Knupp C Loch TP Faisal M. Assessment of cross-protection to heterologous strains of *Flavobacterium psychrophilum* following vaccination with a live-attenuated coldwater disease immersion vaccine. J Fish Dis (2019) 42(1):75–84. doi: 10.1111/jfd.12902 30370695

[19] Vartak A Sucheck SJ. Recent advances in subunit vaccine carriers. Vaccines (Basel) (2016) 4(2):12. doi: 10.3390/vaccines4020012 27104575 PMC4931629

[20] Kim TH Don Hwang S Kim SJ Kim MS Choi HS Han HJ. Efficacy of a recombinant m-like protein, SimA as a subunit vaccine candidate against *Streptococcus parauberis* infection in olive flounder, *Paralichthys olivaceus* . Fish Shellfish Immunol (2022) 131:1092–100. doi: 10.1016/j.fsi.2022.10.009 36257554

[21] Ding Z Wang X Liu Y Zheng Y Li H Zhang M. Dietary mannan oligosaccharides enhance the non-specific immunity, intestinal health, and resistance capacity of juvenile blunt snout bream (*Megalobrama amblycephala*) against *Aeromonas hydrophila* . Front Immunol (2022) 13:863657. doi: 10.3389/fimmu.2022.863657 35784342 PMC9240629

[22] Buchmann K. Introduction to fish parasitological methods: classical and molecular techniques. Frederiksberg, Denmark: Biofolia Publishers (2007). p. 130.

[23] Zhao XL Wu G Chen H Li L Kong XH. Analysis of virulence and immunogenic factors in *Aeromonas hydrophila*: Towards the development of live vaccines. J Fish Dis (2020) 43(7):747–55. doi: 10.1111/jfd.13174 32478415

[24] Wang N Yang Z Zang M Liu Y Lu C. Identification of Omp38 by immunoproteomic analysis and evaluation as a potential vaccine antigen against *Aeromonas hydrophila* in Chinese breams. Fish Shellfish Immunol (2013) 34(1):74–81. doi: 10.1016/j.fsi.2012.10.003 23063539

[25] Gong YX Zhu B Liu GL Liu L Ling F Wang GX. Single-walled carbon nanotubes as delivery vehicles enhance the immunoprotective effects of a recombinant vaccine against *Aeromonas hydrophila* . Fish Shellfish Immunol (2015) 42(1):213–20. doi: 10.1016/j.fsi.2014.11.004 25462556

[26] Abdelhamed H Ibrahim I Nho SW Banes MM Wills RW Karsi A. Evaluation of three recombinant outer membrane proteins, OmpA1, tdr, and TbpA, as potential vaccine antigens against virulent *Aeromonas hydrophila* infection in channel catfish (*Ictalurus punctatus*). Fish Shellfish Immunol (2017) 66:480–6. doi: 10.1016/j.fsi.2017.05.043 28532667

[27] Guo Z Lin Y Wang X Fu Y Lin W Lin X. The protective efficacy of four iron-related recombinant proteins and their single-walled carbon nanotube encapsulated counterparts against *Aeromonas hydrophila* infection in zebrafish. Fish Shellfish Immunol (2018) 82:50–9. doi: 10.1016/j.fsi.2018.08.009 30086377

[28] Cui H Shen X Zheng Y Guo P Gu Z Gao Y. Identification, expression patterns, evolutionary characteristics and recombinant protein activities analysis of *CD209* gene from *Megalobrama amblycephala* . Fish Shellfish Immunol (2022) 126:47–56. doi: 10.1016/j.fsi.2022.04.043 35568142

[29] He Y Ding Z Marana MH Dalsgaard I Rzgar J Heidi M. Immersion vaccines against *Yersinia ruckeri* infection in rainbow trout: Comparative effects of strain differences. J Fish Dis (2021) 44(12):1937–50. doi: 10.1111/jfd.13507 PMC9290694 34392540

[30] Livak KJ Schmittgen TD. Analysis of relative gene expression data using real-time quantitative PCR and the 2(-delta delta C(T)) method. Methods (2001) 25(4):402–8. doi: 10.1006/meth.2001.1262 11846609

[31] Wang QC Ji W Xu Z. Current use and development of fish vaccines in China. Fish Shellfish Immunol (2020) 96:223–34. doi: 10.1016/j.fsi.2019.12.010 31821845

[32] Yun S Jun JW Giri SS Kim HJ Chi C Kim SG. Efficacy of PLGA microparticle-encapsulated formalin-killed *Aeromonas hydrophila* cells as a single-shot vaccine against a. hydrophila infection. Vaccine (2017) 35(32):3959–65. doi: 10.1016/j.vaccine.2017.06.005 28623029

[33] Dubey S Avadhani K Mutalik S Sivadasan SM Maiti B Paul J. *Aeromonas hydrophila* OmpW PLGA nanoparticle oral vaccine shows a dose-dependent protective immunity in rohu (*Labeo rohita*). Vaccines (2016) 4(2):21. doi: 10.3390/vaccines4020021 27258315 PMC4931638

[34] Yadav SK Dash P Sahoo PK Garg LC Dixit A. Recombinant outer membrane protein OmpC induces protective immunity against *Aeromonas hydrophila* infection in *Labeo rohita* . Microb Pathog (2021) 150:104727. doi: 10.1016/j.micpath.2020.104727 33429054

[35] Yadav SK Dash P Sahoo PK Garg LC Dixit A. Modulation of immune response and protective efficacy of recombinant outer-membrane protein f (rOmpF) of *Aeromonas hydrophila* in *Labeo rohita* . Fish Shellfish Immunol (2018) 80:563–72. doi: 10.1016/j.fsi.2018.06.041 29958980

[36] Zhao Z Wang H Zhang D Guan Y Siddiqui SA Feng-Shan X. Oral vaccination with recombinant *Lactobacillus casei* expressing *Aeromonas hydrophila* Aha1 against a. hydrophila infections in common carps. Virulence (2022) 13(1):794–807. doi: 10.1080/21505594.2022.2063484 35499101 PMC9067532

[37] Sughra F Rahman MH Abbas F Altaf I. Evaluation of three alum-precipitated *Aeromonas hydrophila* vaccines administered to *Labeo rohita*, *Cirrhinus mrigala* and *Ctenopharyngodon idella*: Immunokinetics, immersion challenge and histopathology. Braz J Biol (2021) 83:e249913. doi: 10.1590/1519-6984.249913 34550293

[38] Reyes-Becerril M Tovar-Ramírez D Ascencio-Valle F Civera-Cerecedo R Gracia-López V Barbosa-Solomieu V. Effects of dietary supplementation with probiotic live yeast *Debaryomyces hansenii* on the immune and antioxidant systems of leopard grouper *Mycteroperca rosacea* infected with *Aeromonas hydrophila* . Aquac Res (2011) 42(11):1676–86. doi: 10.1111/j.1365-2109.2010.02762.x

[39] Chen G Liu Y Jiang J Jiang W Kuang S Tang L. Effect of dietary arginine on the immune response and gene expression in head kidney and spleen following infection of jian carp with aeromonas hydrophila. Fish Shellfish Immunol (2015) 44(1):195–202. doi: 10.1016/j.fsi.2015.02.027 25721332

[40] Abdelhamed H Nho SW Turaga G Banes MM Karsi A Lawrence ML. Protective efficacy of four recombinant fimbrial proteins of virulent *Aeromonas hydrophila* strain ML09-119 in channel catfish. Vet Microbiol (2016) 197:8–14. doi: 10.1016/j.vetmic.2016.10.026 27938688

[41] Zheng L Feng L Jiang WD Wu P Tang L Kuang SY. Selenium defciency impaired immune function of the immune organs in young grass carp (*Ctenopharyngodon idella*). Fish Shellfish Immunol (2018) 77:53–70. doi: 10.1016/j.fsi.2018.03.024 29559270

[42] Magnadóttir B. Innate immunity of fish (overview). Fish Shellfish Immunol (2006) 20(2):137–51. doi: 10.1016/j.fsi.2004.09.006 15950491

[43] Li C Ren Y Jiang S Zhou S Zhao J Wang R. Effects of dietary supplementation of four strains of lactic acid bacteria on growth, immune-related response and genes expression of the juvenile sea cucumber *Apostichopus japonicus selenka* . Fish Shellfish Immunol (2018) 74:69–75. doi: 10.1016/j.fsi.2017.12.037 29284147

[44] Saurabh S Sahoo PK. Lysozyme: an important defence molecule of fish innate immune system. Aquac Res (2008) 39(3):223–39. doi: 10.1111/j.1365-2109.2007.01883.x

[45] Yu SH Ao J Chen X. Molecular characterization and expression analysis of MHC class II α and β genes in large yellow croaker (*Pseudosciaena crocea*). Mol Biol Rep (2010) 37(3):1295–307. doi: 10.1007/s11033-009-9504-8 19301143

[46] Jørgensen SM Lyng-Syvertsen B Lukacs M Grimholt U Gjøen T. Expression of MHC class I pathway genes in response to infectious salmon anaemia virus in Atlantic salmon (*Salmo salar l.*) cells. Fish Shellfish Immunol (2006) 21(5):548–60. doi: 10.1016/j.fsi.2006.03.004 16772112

[47] Luo W Zhang J Wen JF Liu H Wang WM Gao ZX. Molecular cloning and expression analysis of major histocompatibility complex class I, IIA and IIB genes of blunt snout bream (*Megalobrama amblycephala*). Dev Comp Immunol (2014) 42(2):169–73. doi: 10.1016/j.dci.2013.08.011 23994238

[48] Yamaguchi T Takizawa F Furihata M Soto-Lampe V Dijkstra JM Fischer U. Teleost cytotoxic T cells. Fish Shellfish Immunol (2019) 95:422–39. doi: 10.1016/j.fsi.2019.10.041 31669897

[49] Sukeda M Shiota K Kondo M Nagasawa T Nakao M Somamoto T. Innate cell-mediated cytotoxicity of CD8(+) T cells against the protozoan parasite *Ichthyophthirius multifiliis* in the ginbuna crucian carp, *Carassius auratus langsdorfii* . Dev Comp Immunol (2021) 115:103886. doi: 10.1016/j.dci.2020.103886 33045272

[50] Somamoto T Yoshiura Y Sato A Nakao M Nakanishi T Okamoto N. Expression profiles of TCRβ and CD8α mRNA correlate with virus-specific cell-mediated cytotoxic activity in ginbuna crucian carp. Virology (2006) 348(2):370–7. doi: 10.1016/j.virol.2006.01.019 16497350

[51] Xia H Wu K Liu W Gul Y Wang W Zhang X. Molecular cloning and expression analysis of immunoglobulin m heavy chain gene of blunt snout bream (*Megalobrama amblycephala*). Fish Shellfish Immunol (2014) 40(1):129–35. doi: 10.1016/j.fsi.2014.06.026 24979225

[52] Xia H Wu K Liu W Wang W Zhang X. Spatio-temporal expression of blunt snout bream (*Megalobrama amblycephala*) mIgD and its immune response to *Aeromonas hydrophila* . Cent Eur J Immunol (2015) 40(2):132–41. doi: 10.5114/ceji.2015.52825 PMC4637386 26557025

[53] Xia H Liu W Wu K Wang W Zhang X. sIgZ exhibited maternal transmission in embryonic development and played a prominent role in mucosal immune response of *Megalabrama amblycephala* . Fish Shellfish Immunol (2016) 54:107–17. doi: 10.1016/j.fsi.2016.03.165 27044330

