# Supplementary material for: Immunogenicity and protective efficacy of OmpA subunit vaccine against Aeromonas hydrophila infection in Megalobrama amblycephala: An effective alternative to the inactivated vaccine
Source: Front Immunol. 2023 Mar 9;14:1133742. doi: 10.3389/fimmu.2023.1133742 (PMC10034085; doi:10.3389/fimmu.2023.1133742)
Supplement: Supplementary file 1 [file DataSheet_1.zip › Supplementary files 23.3.4/Supplementary files 23.3.4.docx]

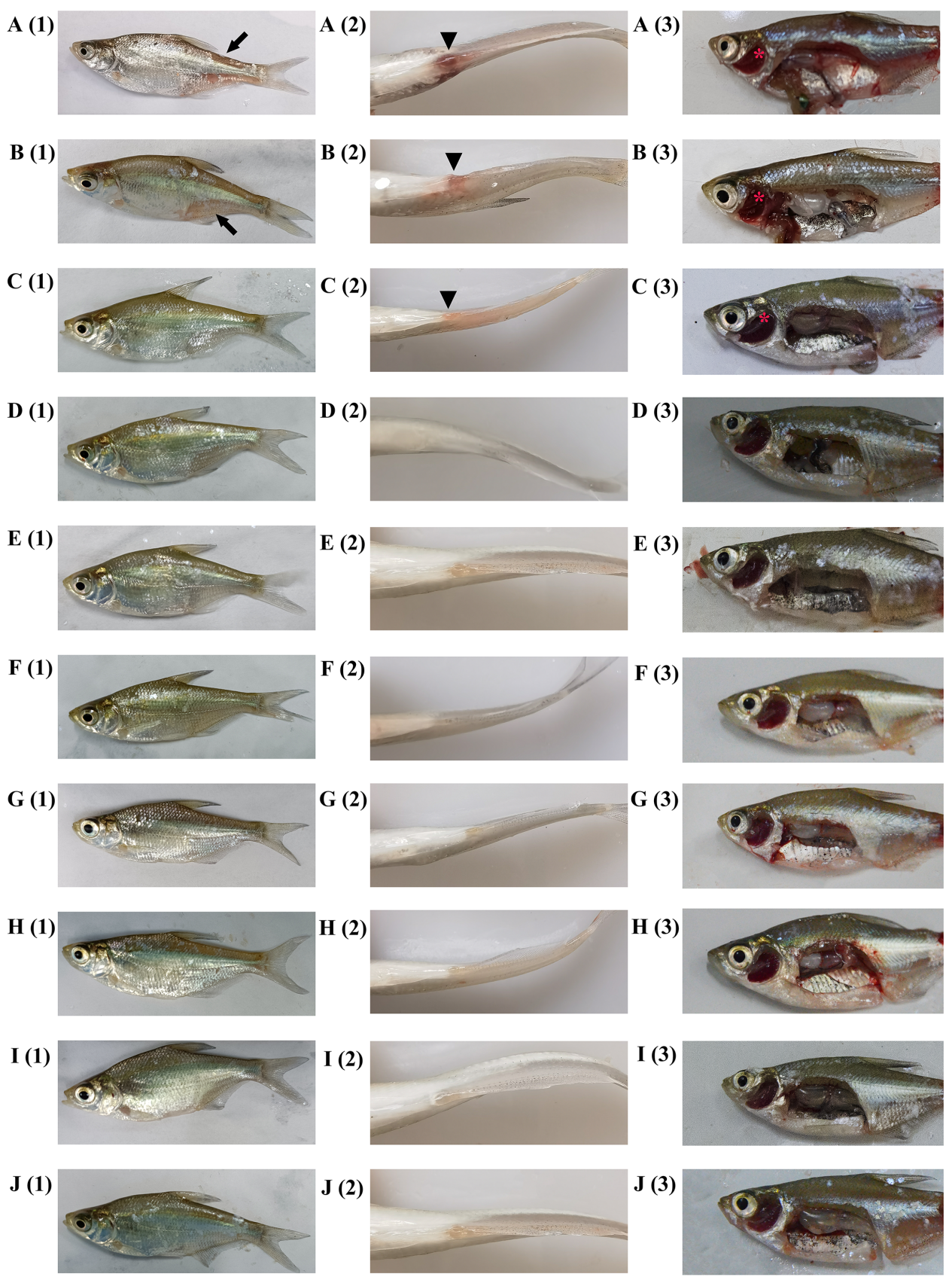


**Supplemental Fig. 1.** Clinical symptoms of *Megalobrama amblycephala* infected by *Aeromonas hydrophila.*

(A) and (B) were control group at 1 and 3 dpi, respectively. (C) and (D) were inactivated vaccine L group at 1 and 3 dpi, respectively. (E) and (F) were inactivated vaccine H group at 1 and 3 dpi, respectively. (G) and (H) were OmpA vaccine L group at 1 and 3 dpi, respectively. (I) and (J) were OmpA vaccine H group at 1 and 3 dpi, respectively. Arrow: congestion of fish body; triangle: swelling around the anus; asterisk: hemorrhage and necrosis of gills. (1), (2) and (3) were images that photographed from different angles at the same time point.


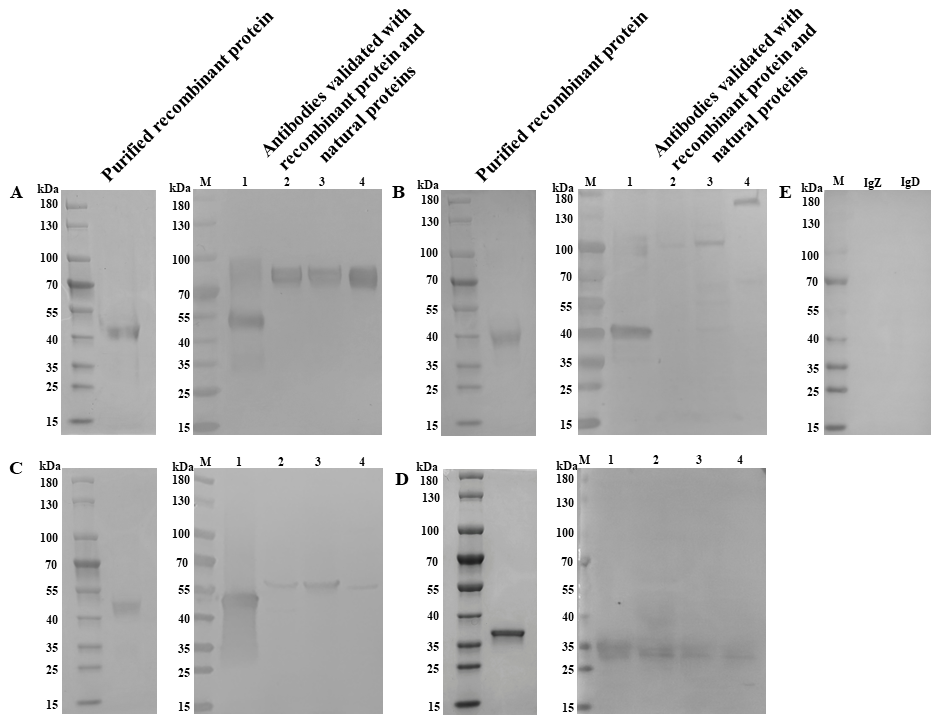


**Supplemental Fig. 2.** Detection of purified recombinant proteins and prepared antibodies.

(A-D) Purified recombinant proteins were detected by SDS-PAGE analysis, and the specificity of prepared antibodies were verified by western blotting with recombinant proteins and natural proteins of *Megalobrama amblycephala.* A-D was IgM, IgD, IgZ and CD8, respectively. M: Marker; 1: recombinant proteins; 2: liver; 3:gill; 4: serum. (E) Verification of the cross-reactions between the recombinant IgD/IgZ proteins and anti-IgM antibody by western blotting analysis. M: Marker.

**
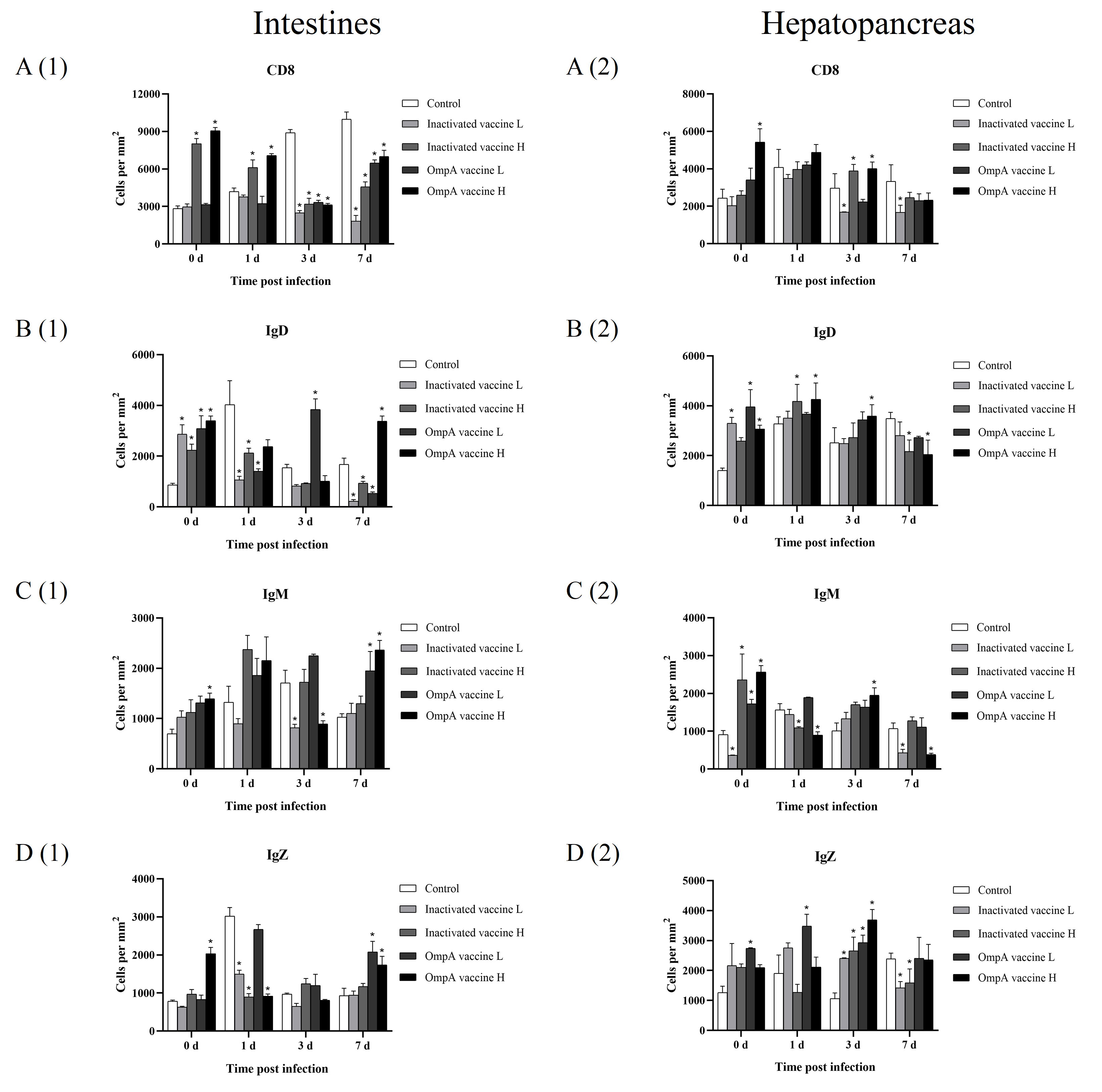
**

**Supplemental Fig. 3.** The number of positive cells in the intestines and hepatopancreas of juvenile *M. amblycephala* post infection.


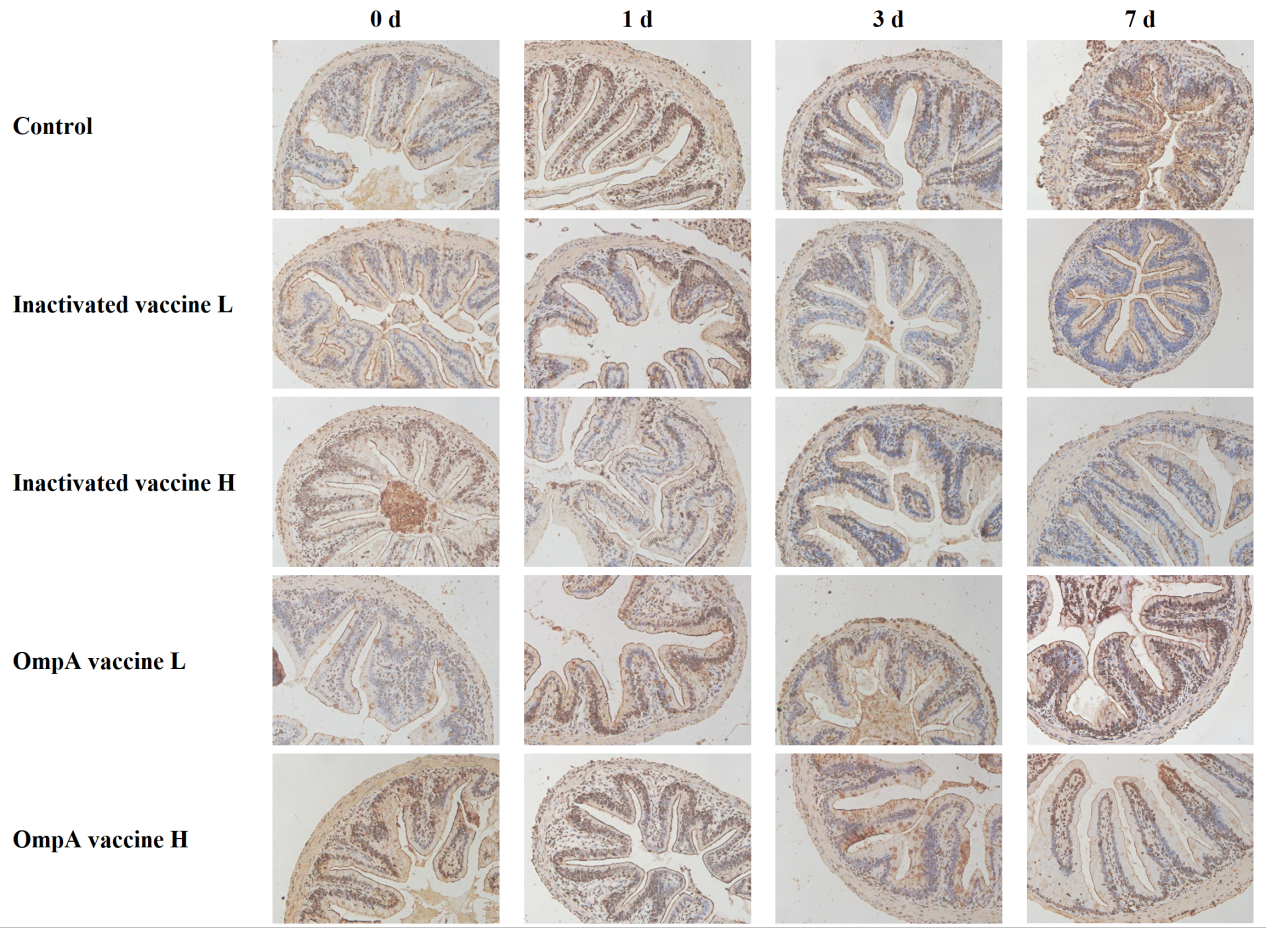


**Supplemental Fig. 4A.** Positive cells to anti-CD8 antibody in the intestines that detected by IHC.


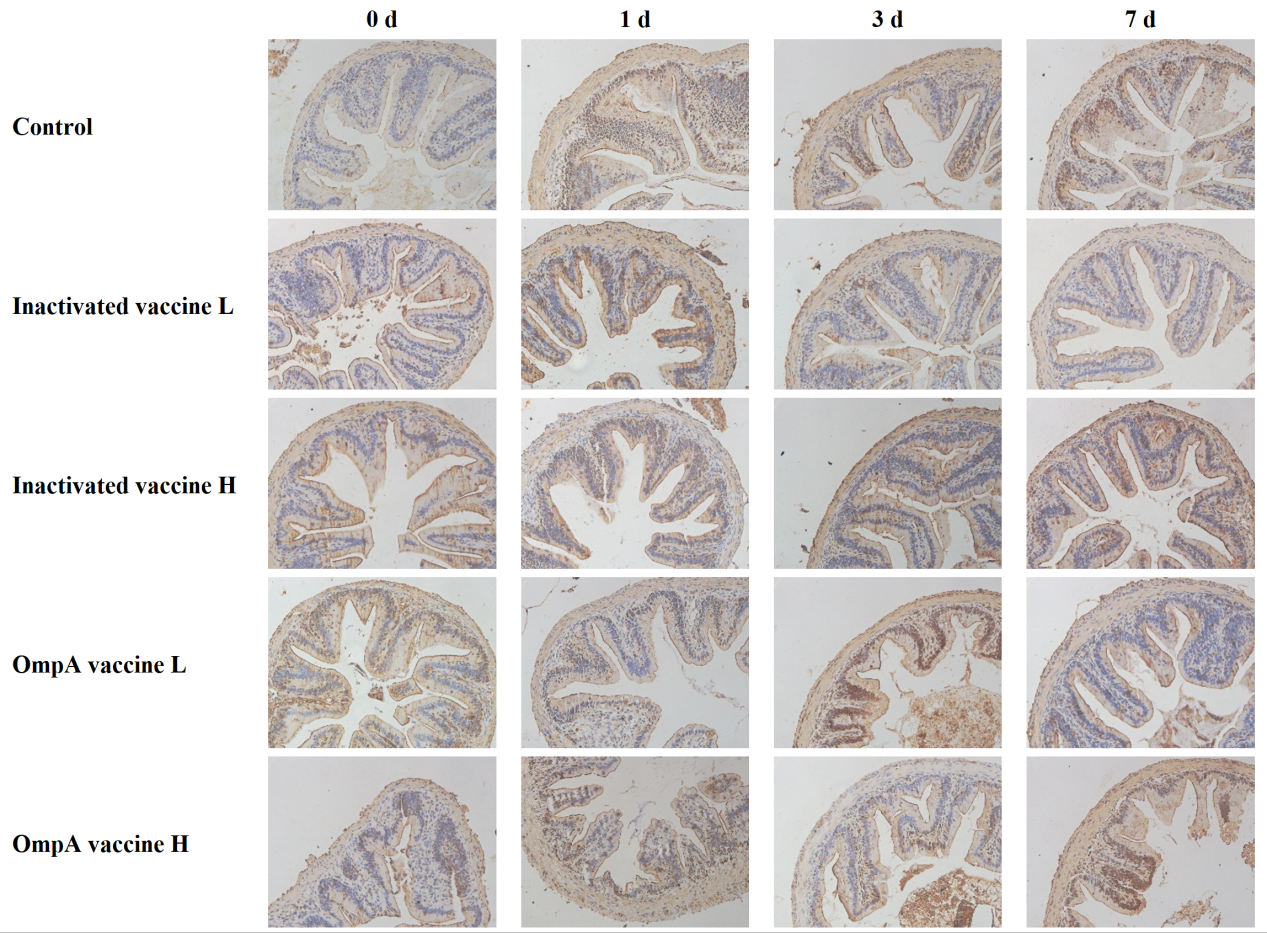


**Supplemental Fig. 4B.** Positive cells to anti-IgD antibody in the intestines that detected by IHC.


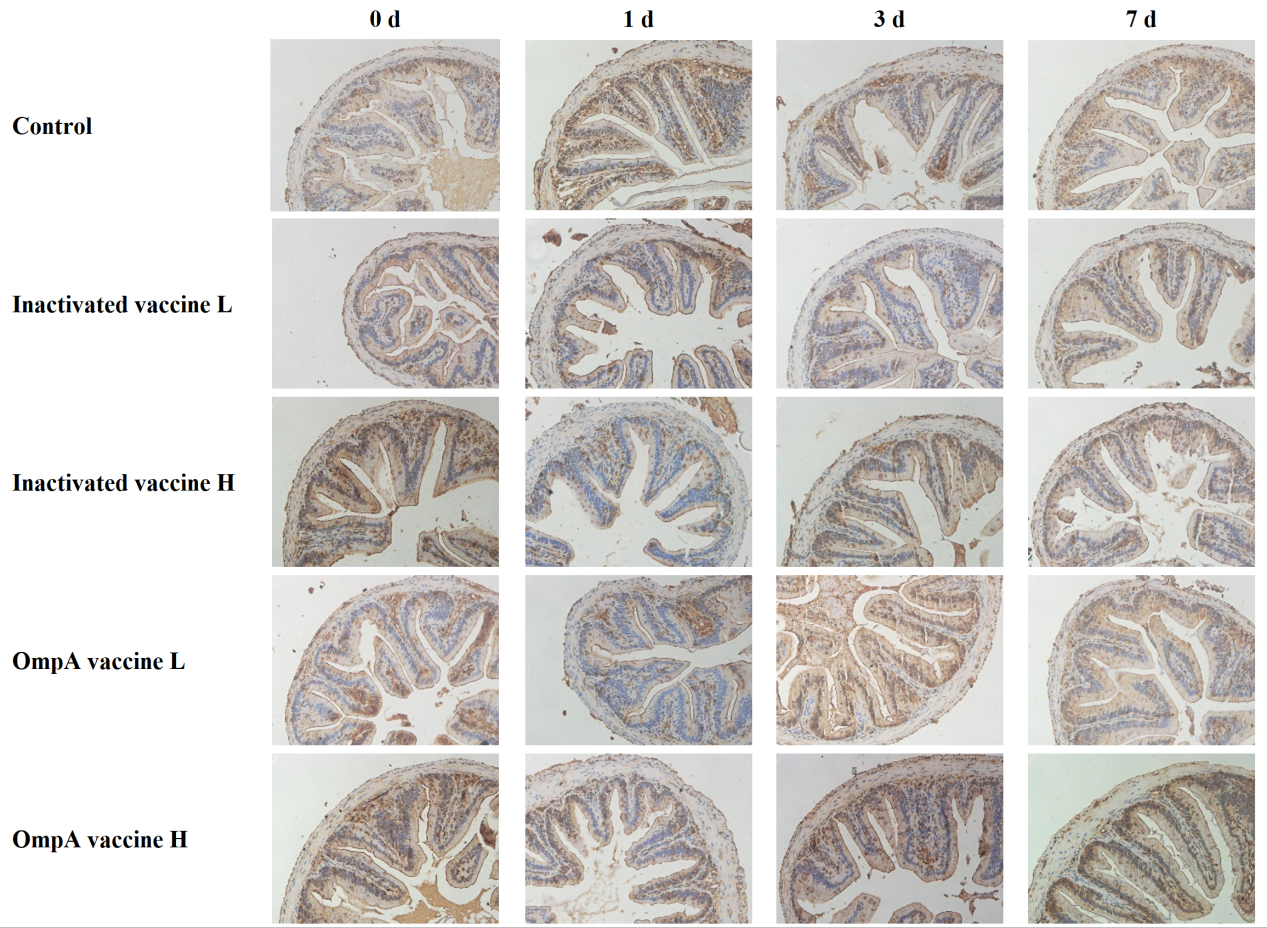


**Supplemental Fig. 4C.** Positive cells to anti-IgM antibody in the intestines that detected by IHC.


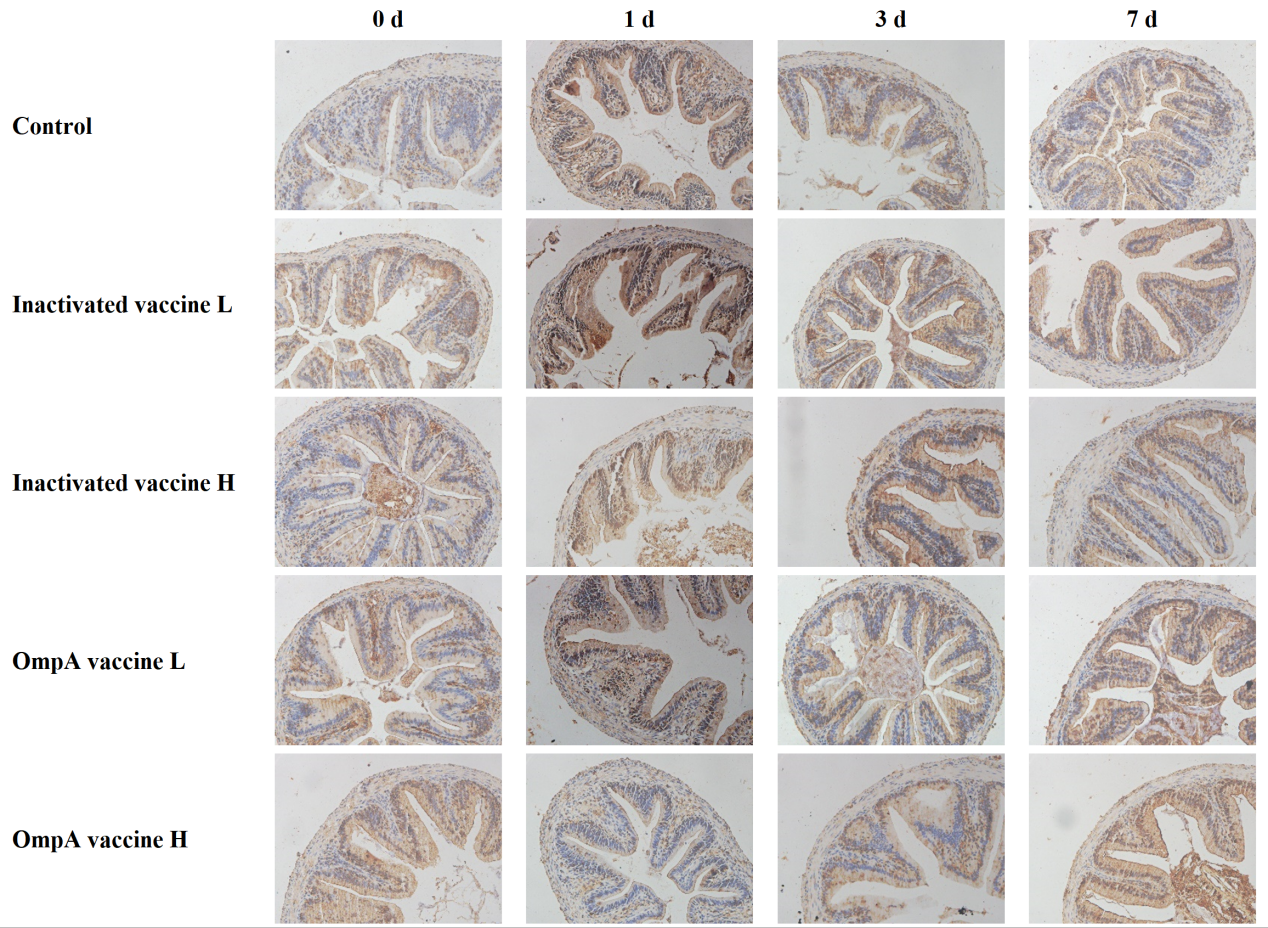


**Supplemental Fig. 4D.** Positive cells to anti-IgZ antibody in the intestines that detected by IHC.


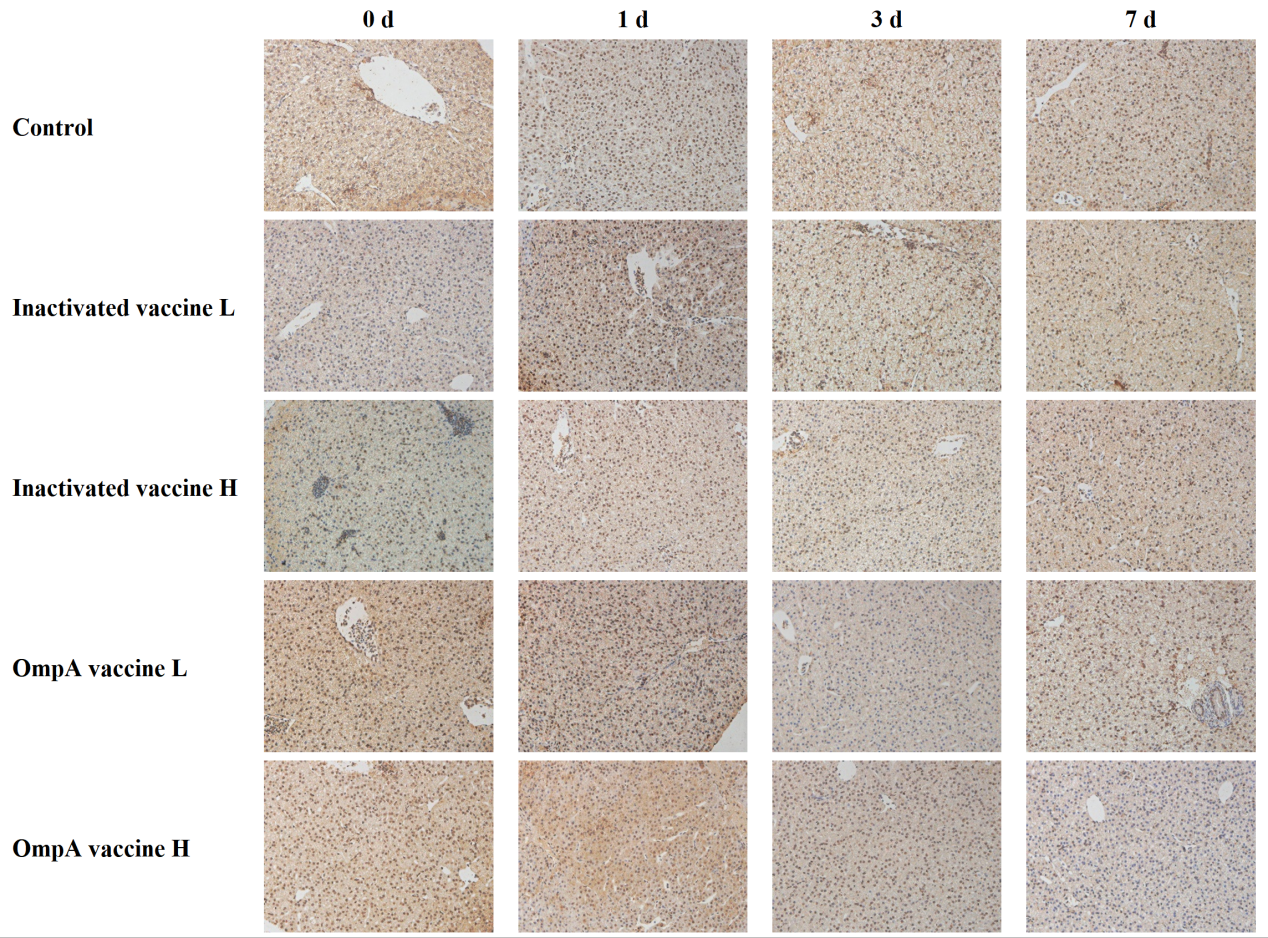


**Supplemental Fig. 5A.** Positive cells to anti-CD8 antibody in the hepatopancreas that detected by IHC.


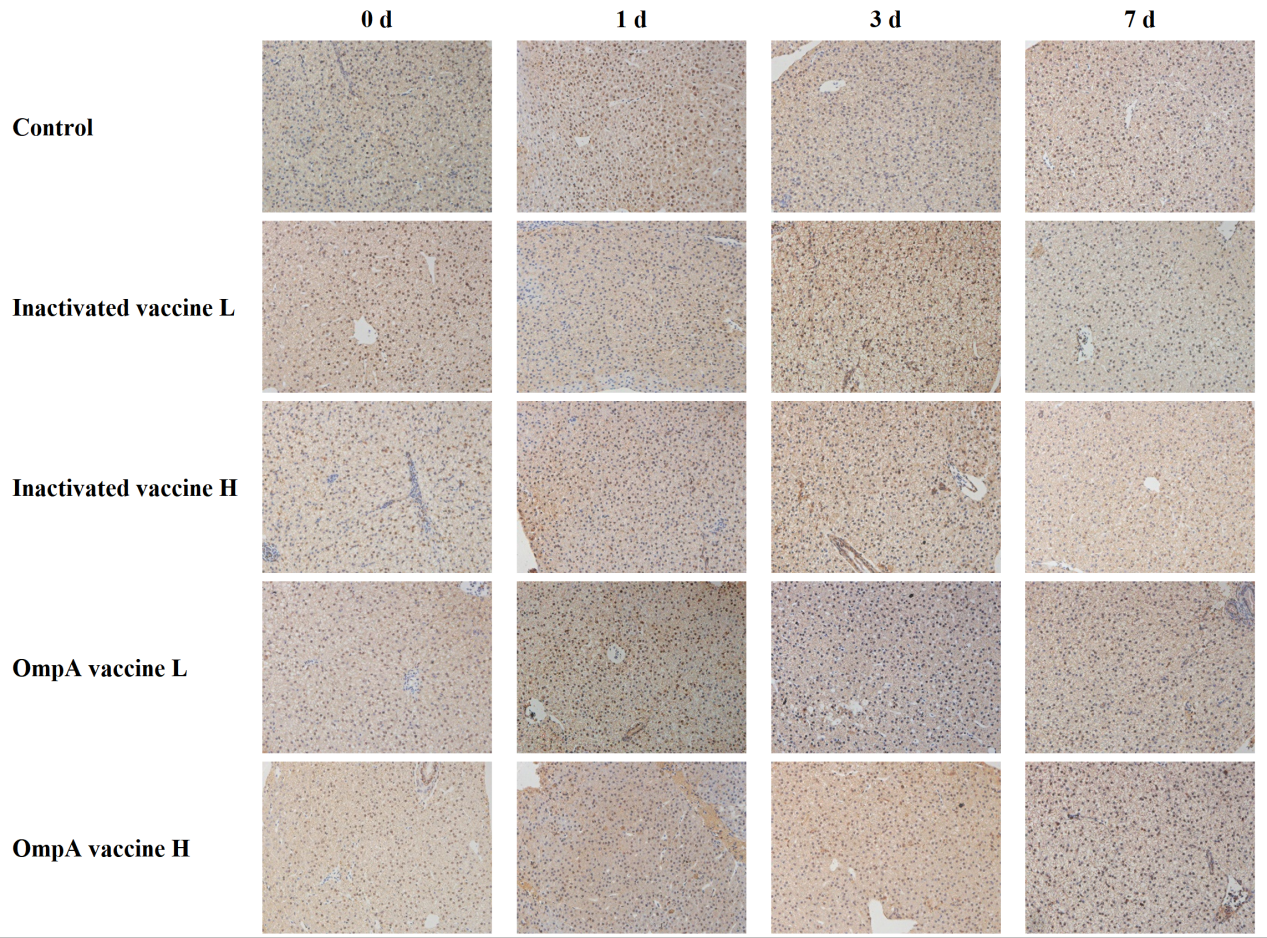


**Supplemental Fig. 5B.** Positive cells to anti-IgD antibody in the hepatopancreas that detected by IHC.


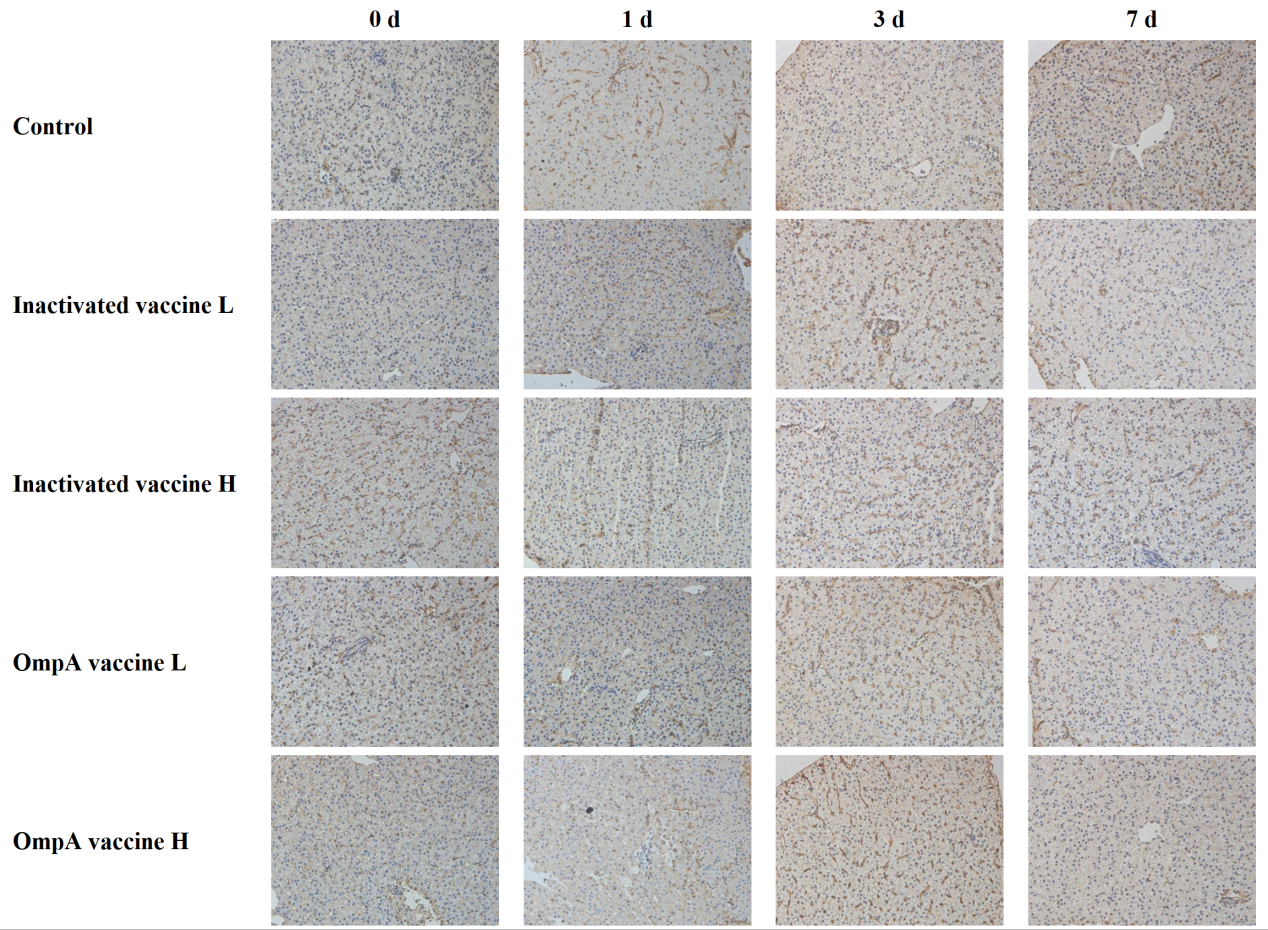


**Supplemental Fig. 5C.** Positive cells to anti-IgM antibody in the hepatopancreas that detected by IHC.


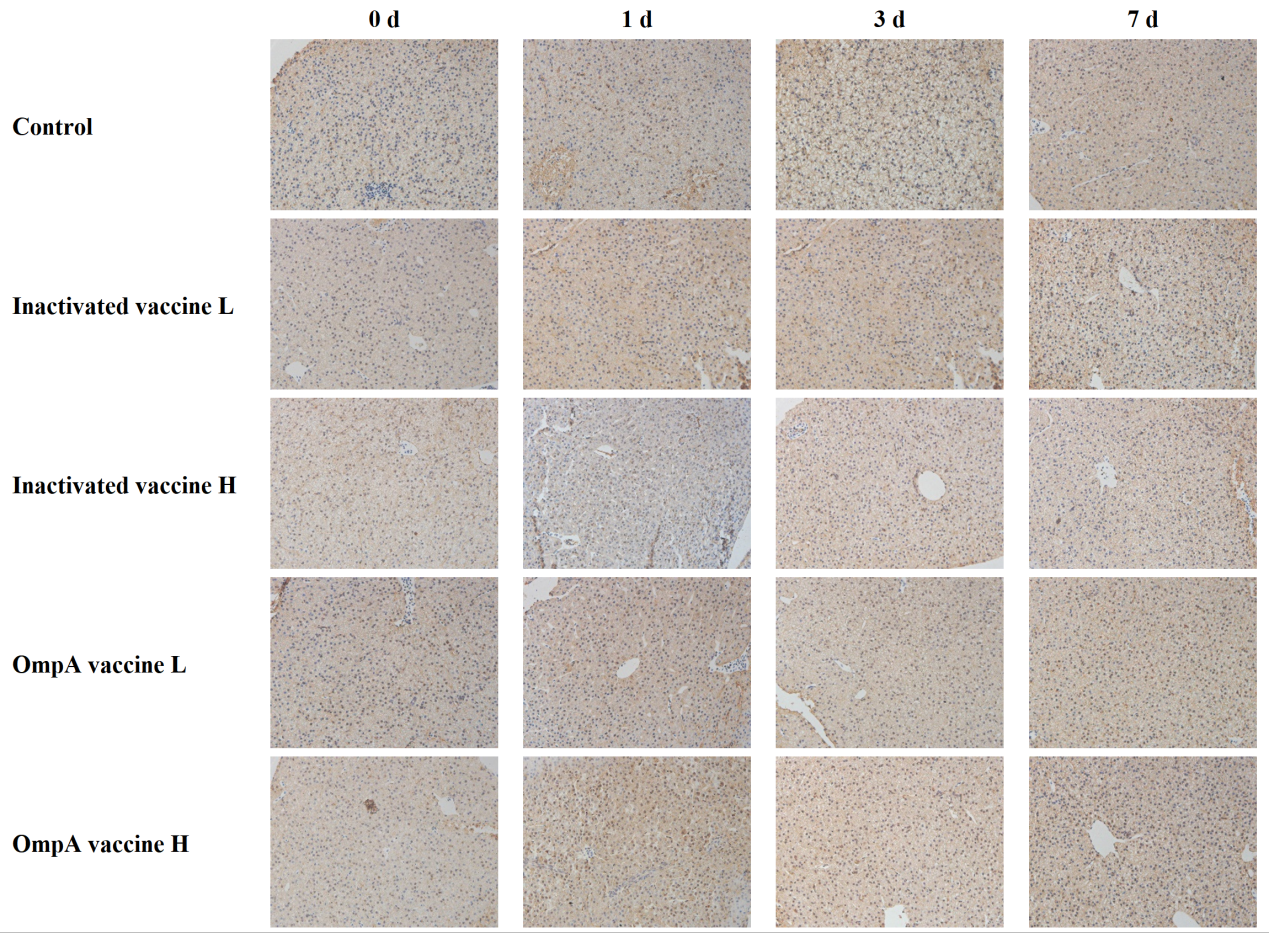


**Supplemental Fig. 5D.** Positive cells to anti-IgZ antibody in the hepatopancreas that detected by IHC.

**
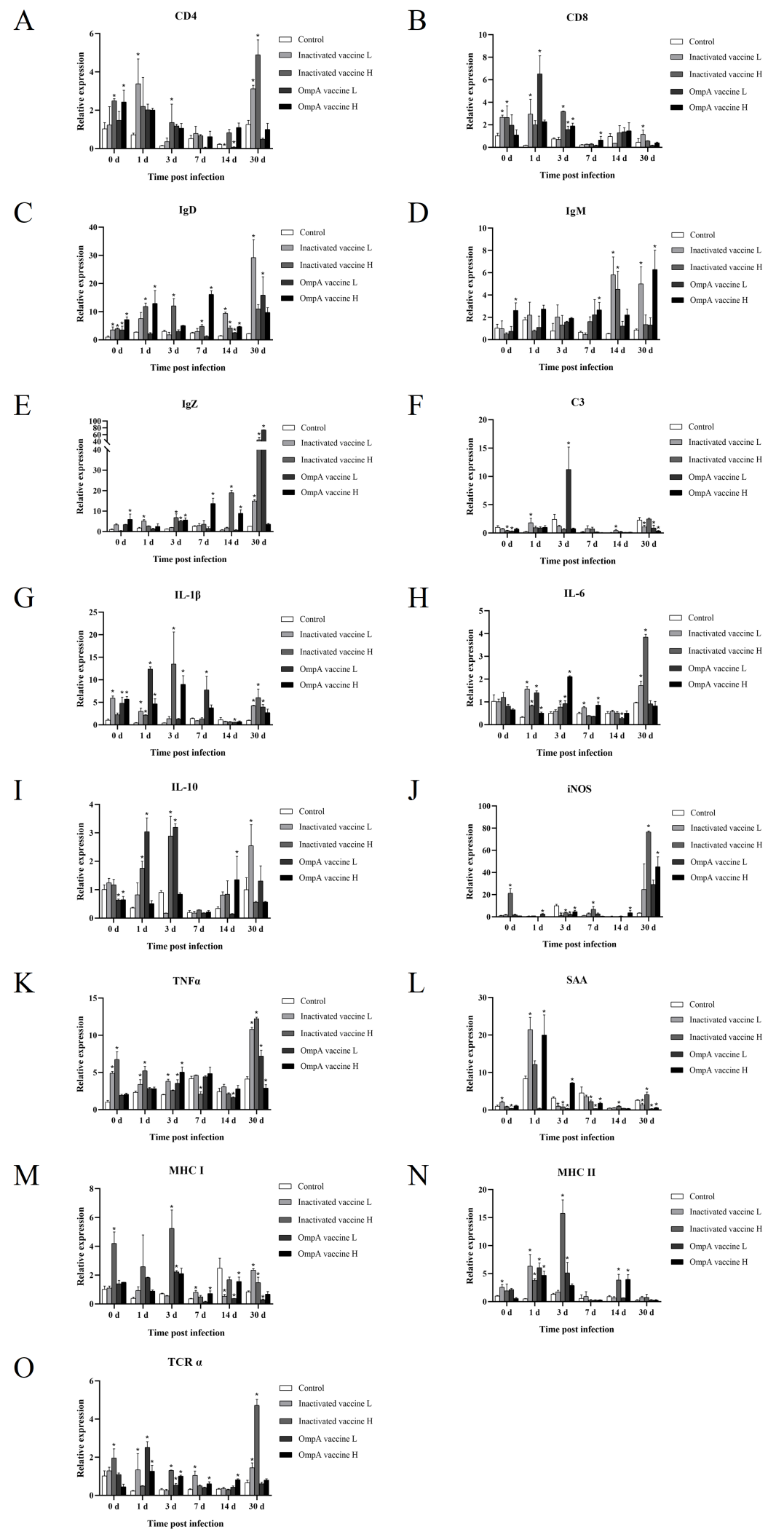
**

**Supplemental Fig. 6A.** Expression of immune related genes in the intestines of *M. amblycephala*.

**
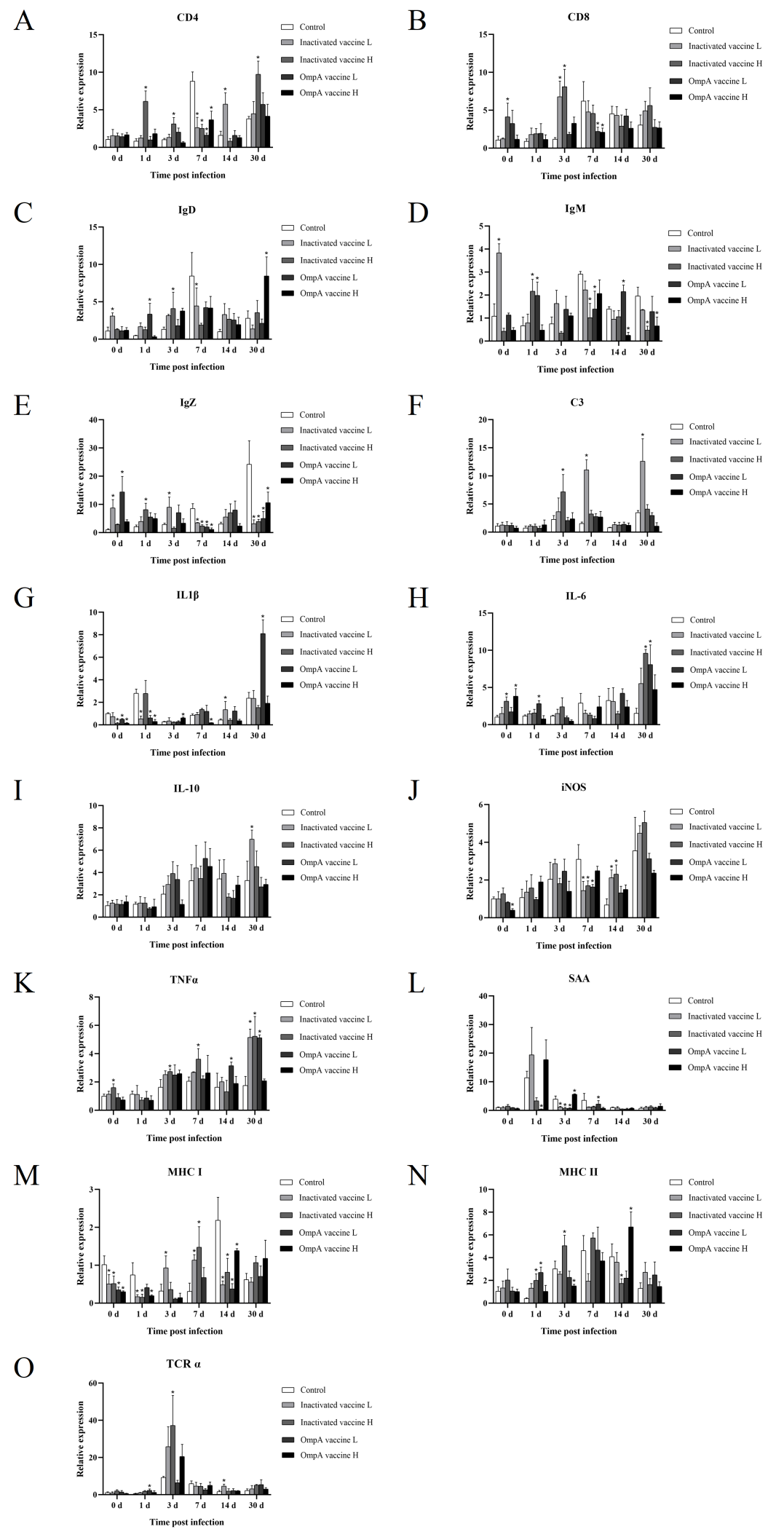
**

**Supplemental Fig. 6B.** Expression of immune related genes in the gills of *M. amblycephala*.

**
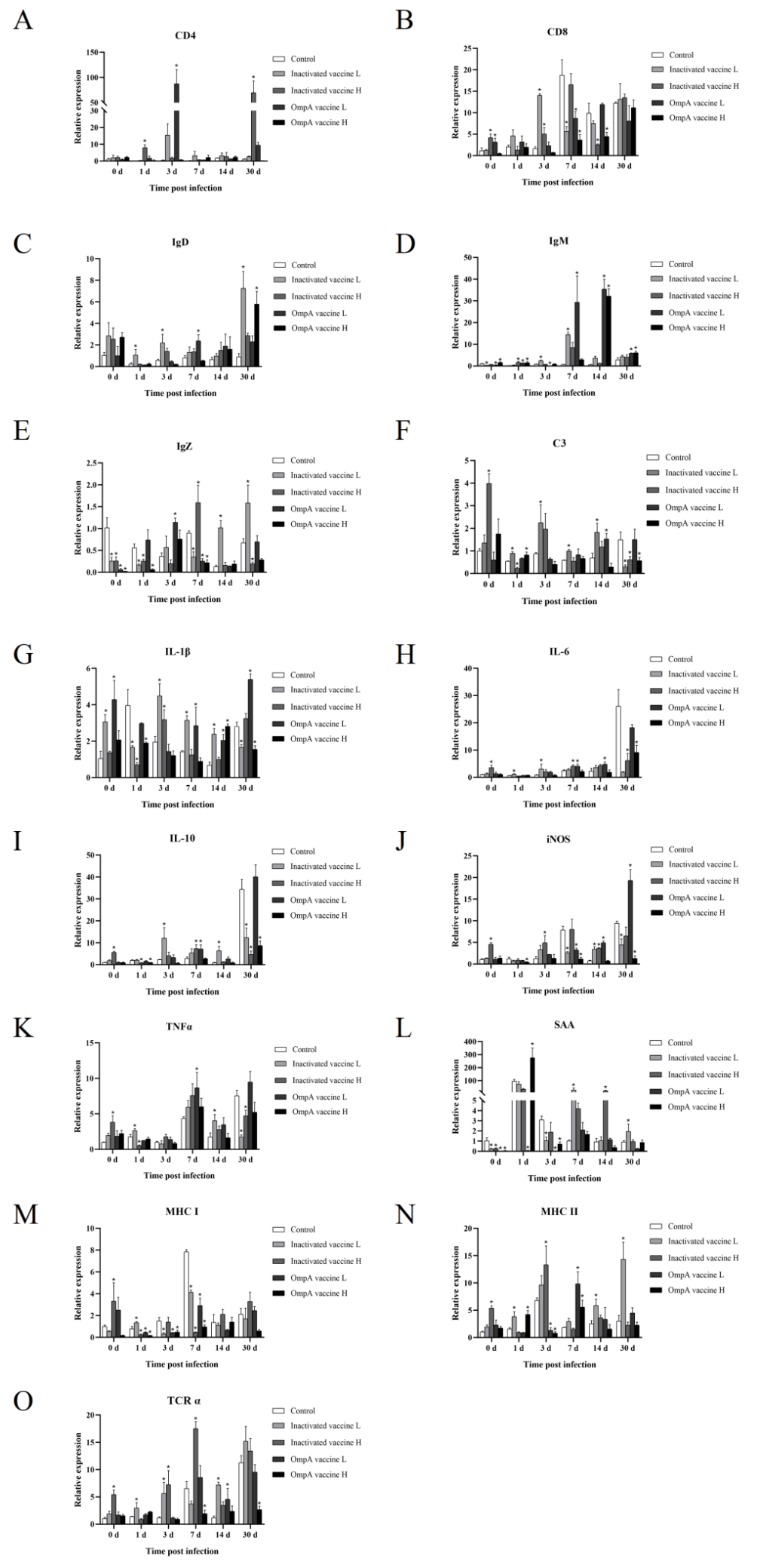
**

**Supplemental Fig. 6C.** Expression of immune related genes in the hepatopancreas of *M. amblycephala*.

**Supplemental Table 1.** Primers used for PCR in the present study.

| **Primers** | **Sequences (5’-3’)** |
| --- | --- |
| OmpA-F | CCATGGCTGACGACATCTACTTCGGTGCC |
| OmpA-R | CTCGAGCTTCTGAACTTCTTGTACGCCAGA |
| q*SAA*-F | GTGCTGGTGCTGGGTTTGGT |
| q*SAA*-R | GCCTTCCTCATATCCTGGTAGGC |
| q*iNOS*-F | ATTCAAGGGCAGCTTCCAGG |
| q*iNOS*-R | CAGGGGCAAAGTTTAAGGGC |
| q*IL1β*-F | CGATAAGACCAGCACGACCTT |
| q*IL1β*-R | GTTTCCGTCTCTCAGCGTCA |
| q*IL6*-F | AAGACAACCGCACACTCGAT |
| q*IL6*-R | CTGGGTCTCTTCACGCCTTT |
| q*IL10*-F | GTGTTTTCGGGTGCAAGTGG |
| q*IL10*-R | ATGAACGAGATCCTGCGCTT |
| q*TNFα*-F | TGATGACGGCATTTACTTCG |
| q*TNFα*-R | CCTCCATAGGAATCAGAATAGC |
| q*C3*-F | ATGGACTTTCACTCGATCCAACA |
| q*C3*-R | AACTGCTTCTCCATCTTCACACT |
| q*MHC I*-F | TTCCAGACGACGAGCACCAT |
| q*MHC I*-R | ACACCACATACCCAGCGACA |
| q*MHC IIβ*-F | CTTCTACCCACCCCAGATCC |
| q*MHC IIβ*-R | GTGATCCACAGCACAGGAGA |
| q*CD4*-F | GAGACGAATTTGATGGTTGTGGC |
| q*CD4*-R | GCTGCTTTGATTGCTGGGAAGT |
| q*CD8α*-F | TTCACTGCGACCCTAAACCG |
| q*CD8α*-R | TTAATGTCCAAGCGTACCTTACCA |
| q*TCRα*-F | TGGCAACTGACTTCACGAAC |
| q*TCRα*-R | CAAAAGAGCCCCAGTGACAT |
| q*IgM*-F | GGAGCAACGGCACAGTAT |
| q*IgM*-R | ATCAGCAAGCCAAGACAC |
| q*IgD*-F | TGGCTGCTTGGAACGACT |
| q*IgD*-R | ATGTTATGCGACTGGGTA |
| q*IgZ*-F | CCAAAGTAAACCGAAGTG |
| q*IgZ*-R | AAAGTGATAGAGGCAGAAC |
| q*16S rRNA*-F | GGGAGTGCCTTCGGGAATCAGA |
| q*16S rRNA*-R | TCACCGCAACATTCTGATTTG |
| q*GAPDH*-F | TGCCGGCATCTCCCTCAA |
| q*GAPDH*-R | TCAGCAACACGGTGGCTGTAG |
