# Supplementary material for: Immunogenicity and protective efficacy of OmpA subunit vaccine against Aeromonas hydrophila infection in Megalobrama amblycephala: An effective alternative to the inactivated vaccine
Source: Front Immunol. 2023 Mar 9;14:1133742. doi: 10.3389/fimmu.2023.1133742 (PMC10034085; doi:10.3389/fimmu.2023.1133742)
Supplement: Supplementary file 1 [file DataSheet_1.zip › Supplementary files 23.3.4/Supplementary files 23.3.4.pdf]

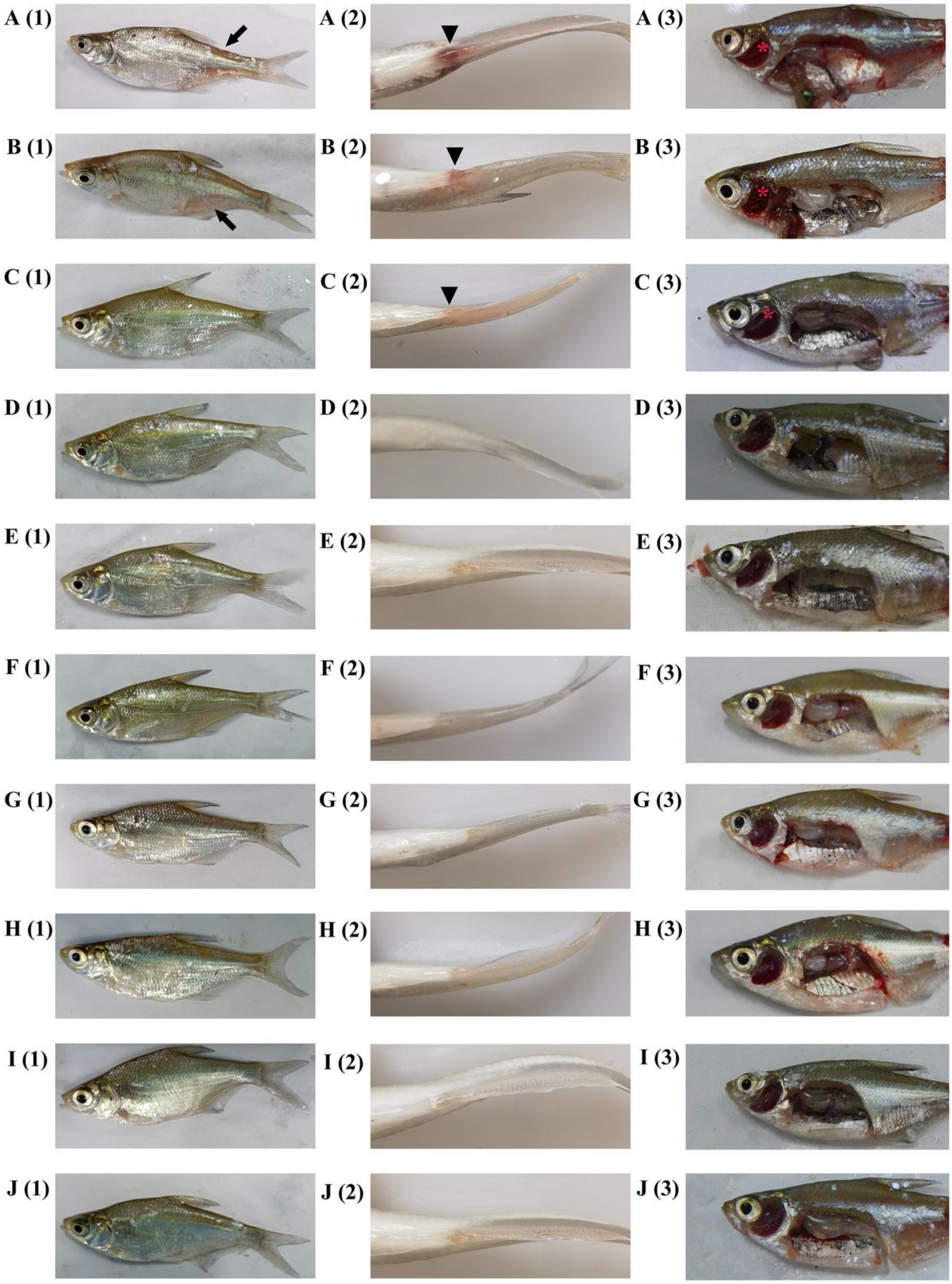

Supplemental Fig. 1. Clinical symptoms of *Megalobrama amblycephala* infected by *Aeromonas hydrophila.*

(A) and (B) were control group at 1 and 3 dpi, respectively. (C) and (D) were inactivated vaccine L group at 1 and 3 dpi, respectively. (E) and (F) were inactivated vaccine H group at 1 and 3 dpi, respectively. (G) and (H) were OmpA vaccine L group at 1 and 3 dpi, respectively. (I) and (J) were OmpA vaccine H group at 1 and 3 dpi, respectively. Arrow: congestion of fish body; triangle: swelling around the anus; asterisk: hemorrhage and necrosis of gills. (1), (2) and (3) were images that photographed from different angles at the same time point.

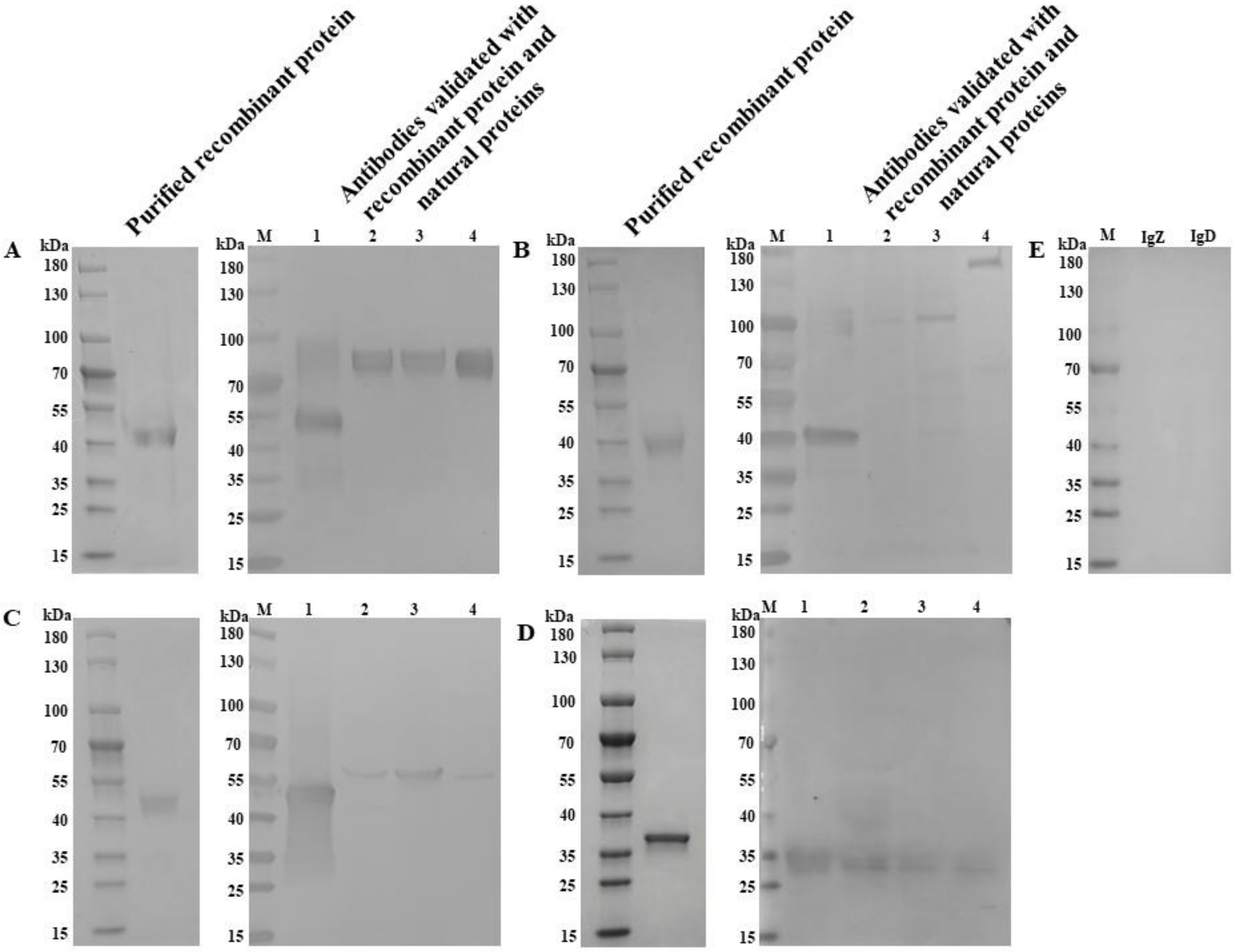

Supplemental Fig. 2. Detection of purified recombinant proteins and prepared antibodies. (A-D) Purified recombinant proteins were detected by SDS-PAGE analysis, and the specificity of prepared antibodies were verified by western blotting with recombinant proteins and natural proteins of *Megalobrama amblycephala.* A-D was IgM, IgD, IgZ and CD8, respectively. M: Marker; 1: recombinant proteins; 2: liver; 3:gill; 4: serum. (E) Verification of the cross-reactions between the recombinant IgD/IgZ proteins and anti-IgM antibody by western blotting analysis. M: Marker.

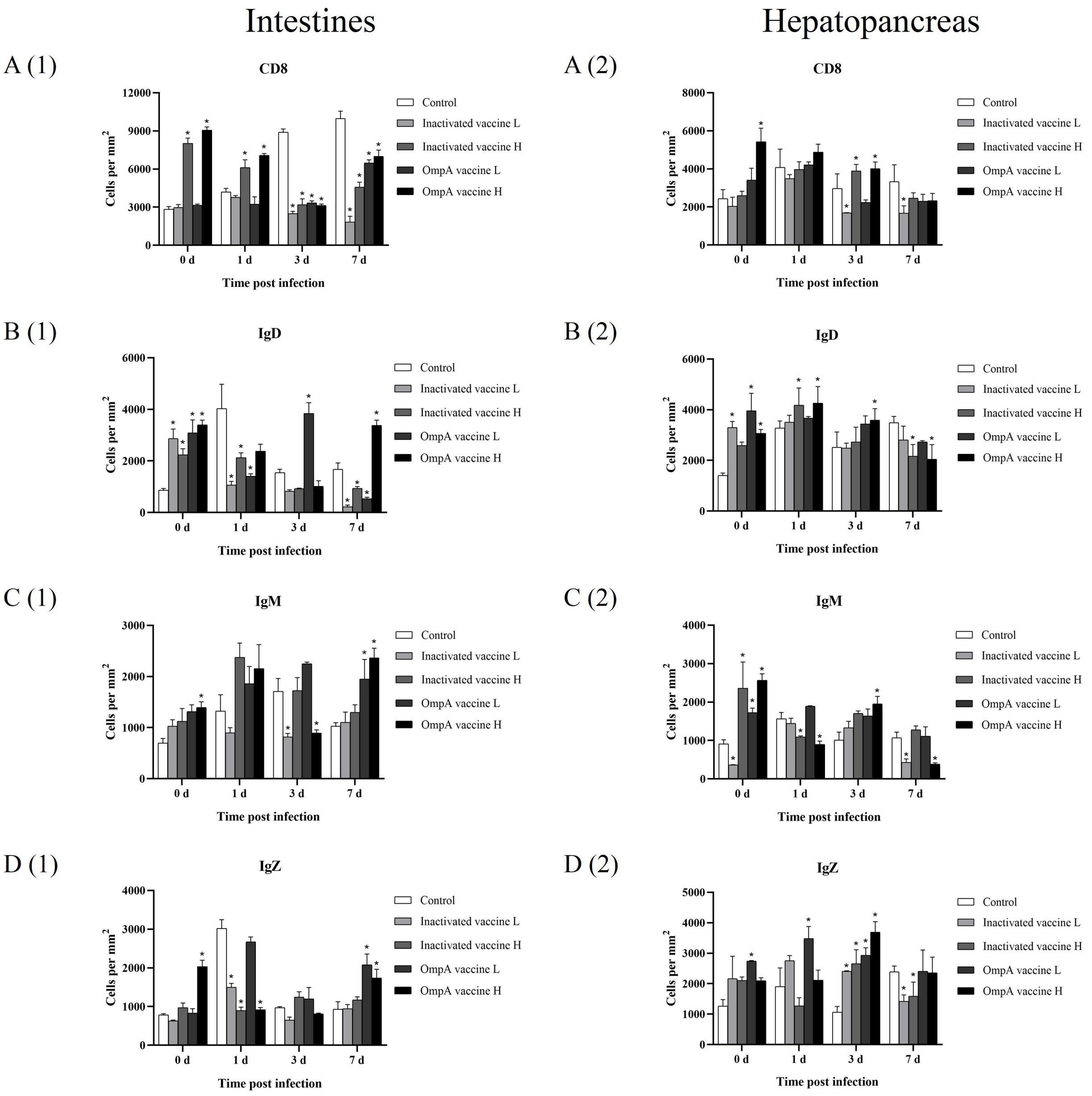

Supplemental Fig. 3. The number of positive cells in the intestines and hepatopancreas of juvenile *M. amblycephala* post infection.

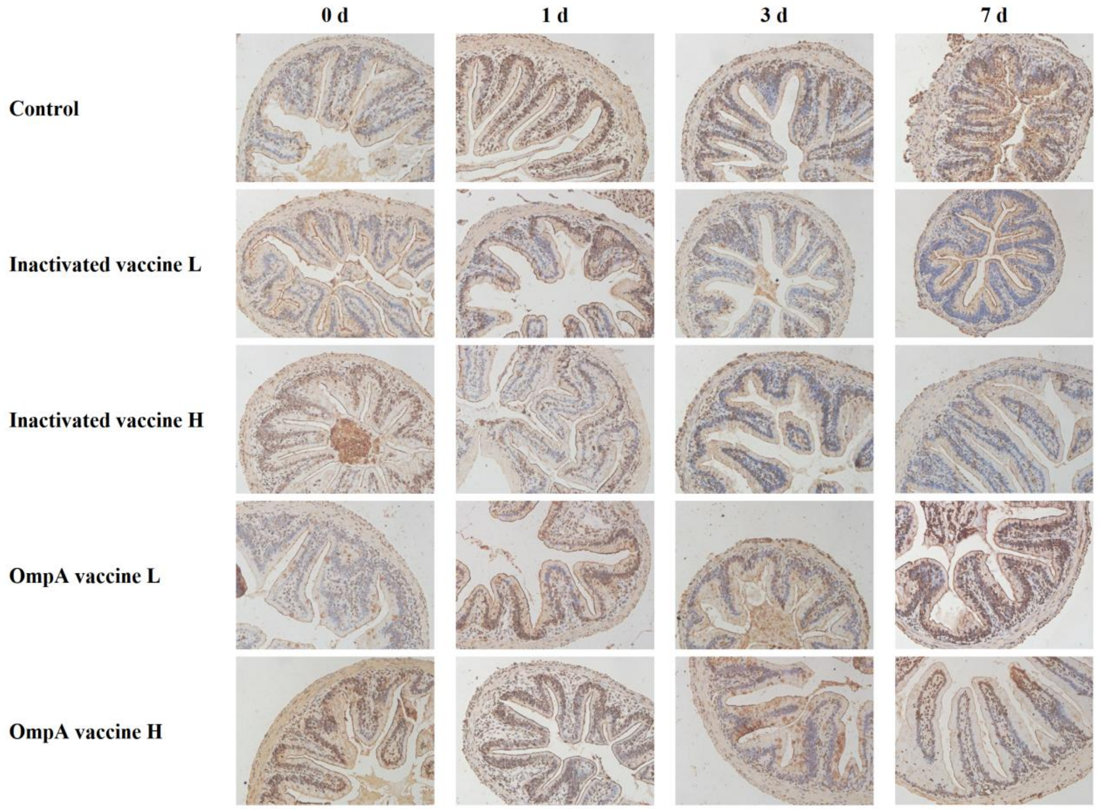

Supplemental Fig. 4A. Positive cells to anti-CD8 antibody in the intestines that detected by IHC.

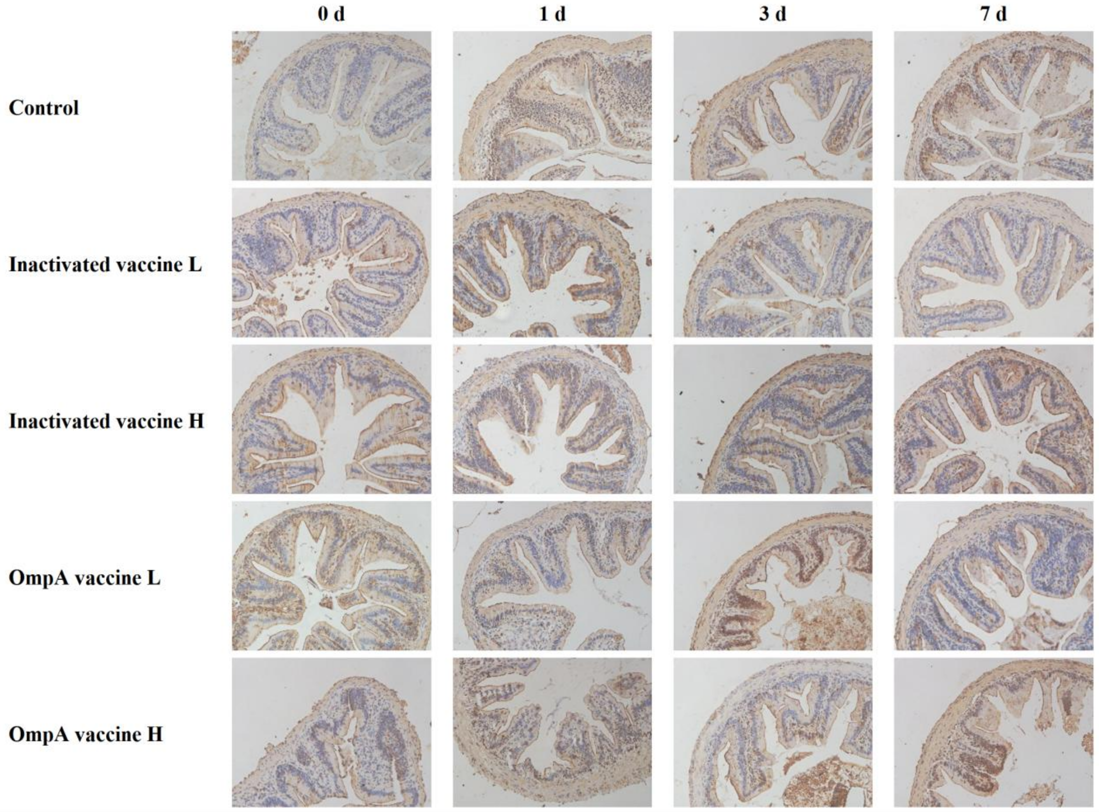

Supplemental Fig. 4B. Positive cells to anti-IgD antibody in the intestines that detected by IHC.

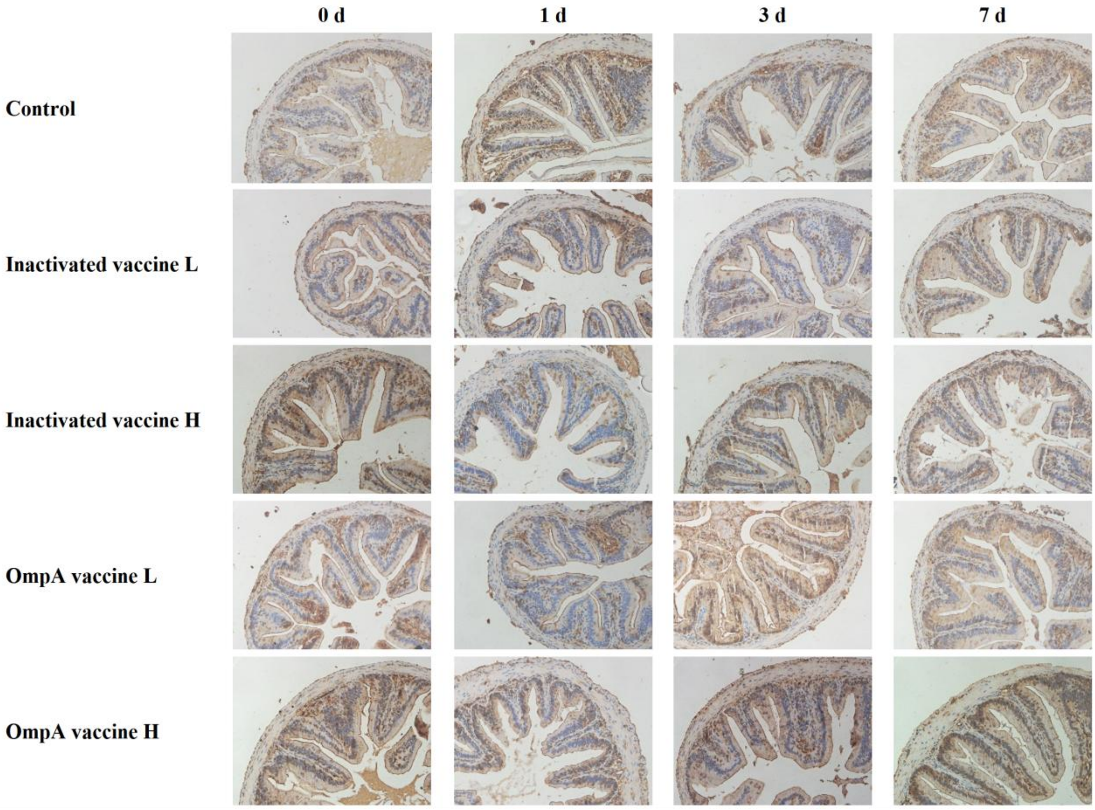

Supplemental Fig. 4C. Positive cells to anti-IgM antibody in the intestines that detected by IHC.

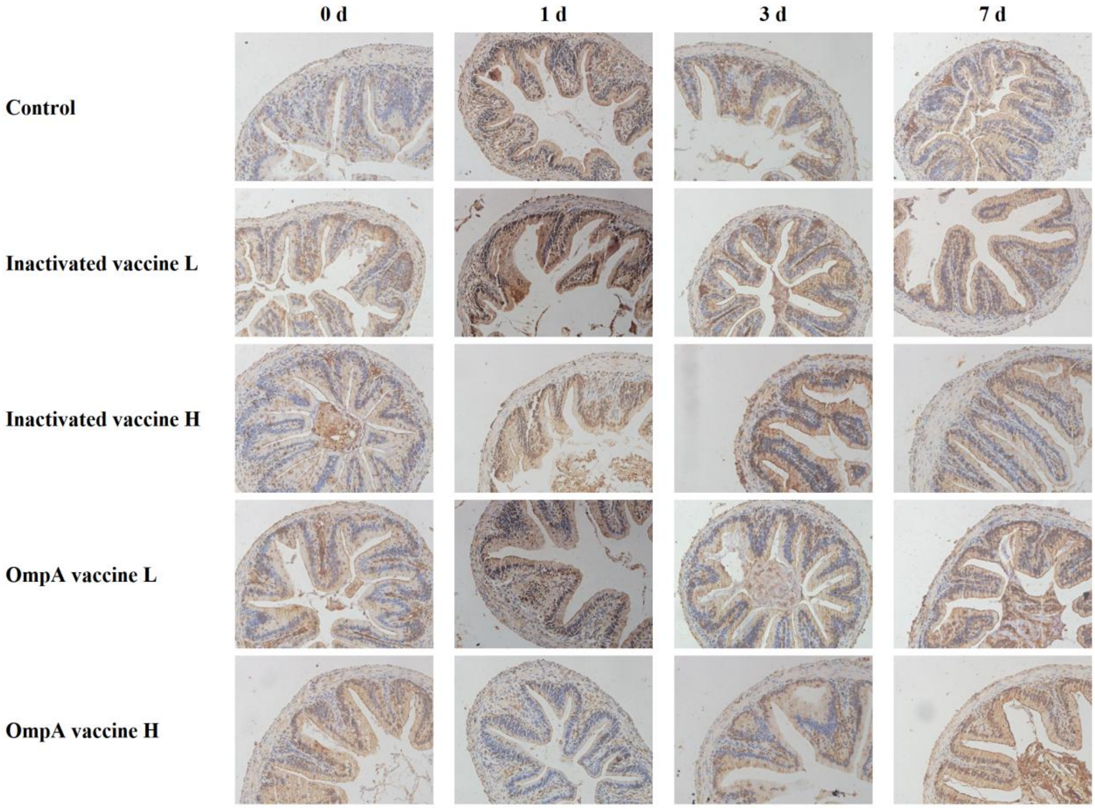

Supplemental Fig. 4D. Positive cells to anti-IgZ antibody in the intestines that detected by IHC.

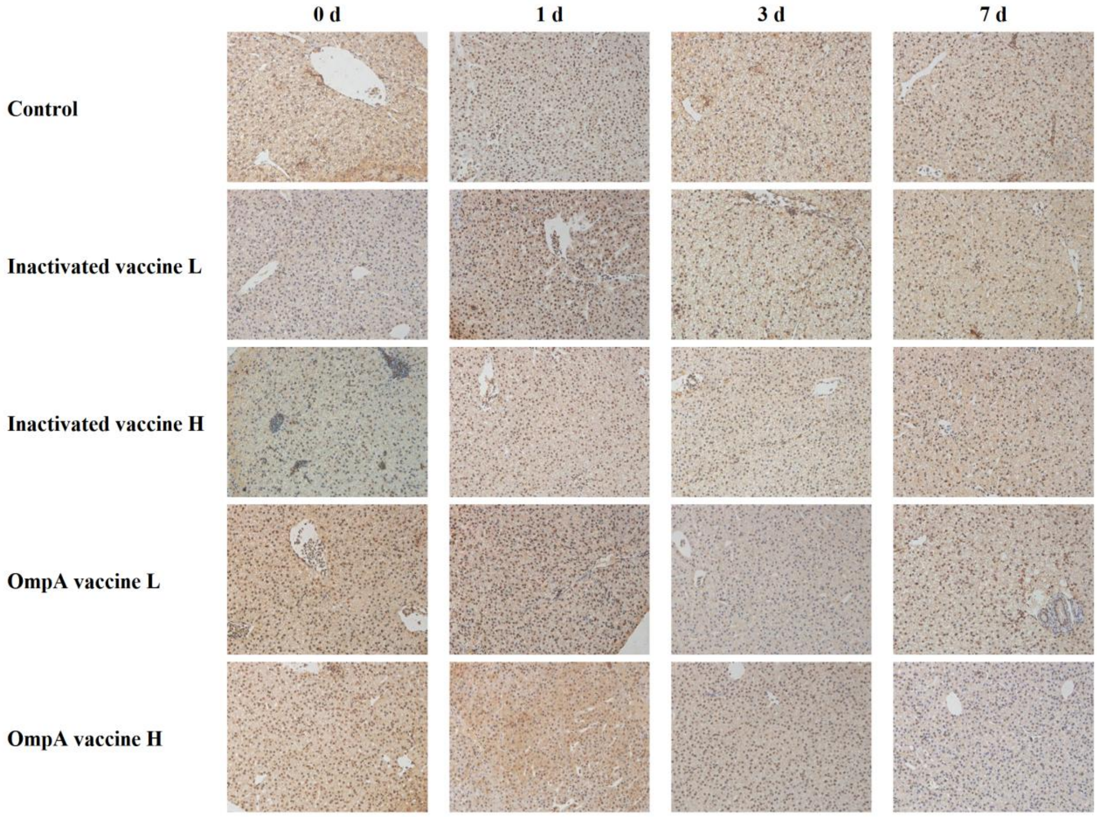

Supplemental Fig. 5A. Positive cells to anti-CD8 antibody in the hepatopancreas that detected by IHC.

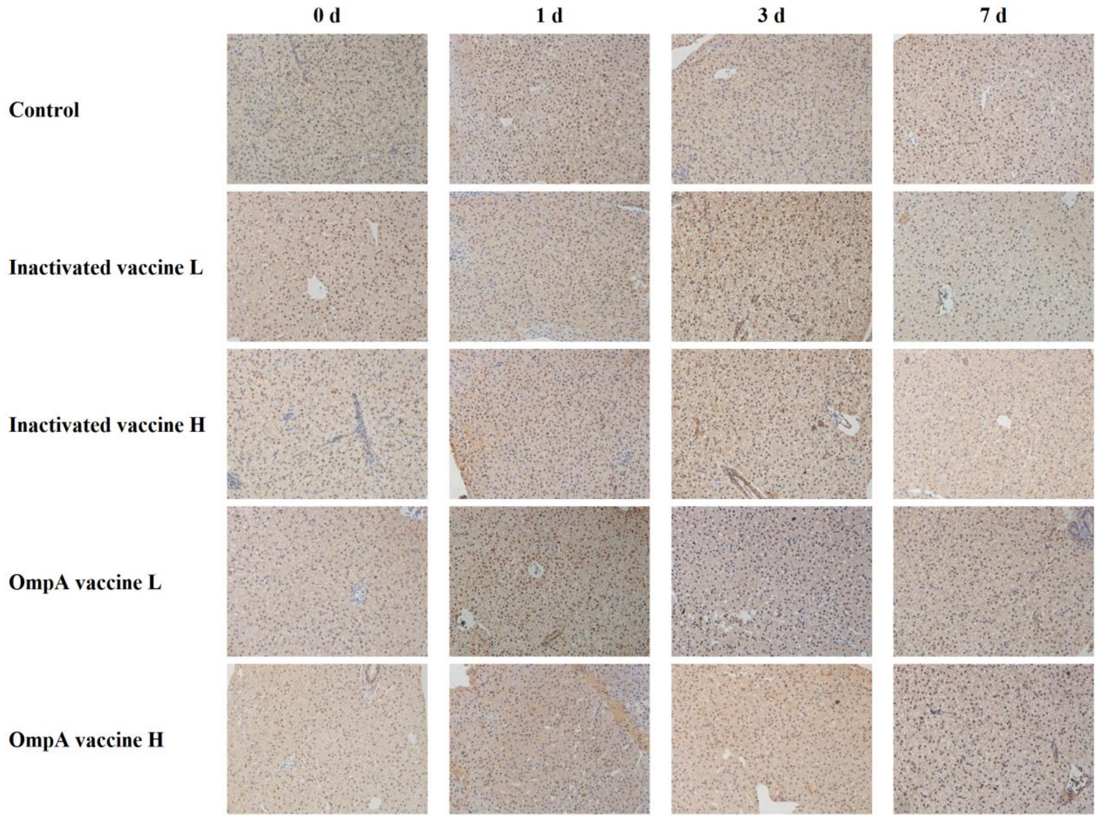

Supplemental Fig. 5B. Positive cells to anti-IgD antibody in the hepatopancreas that detected by IHC.

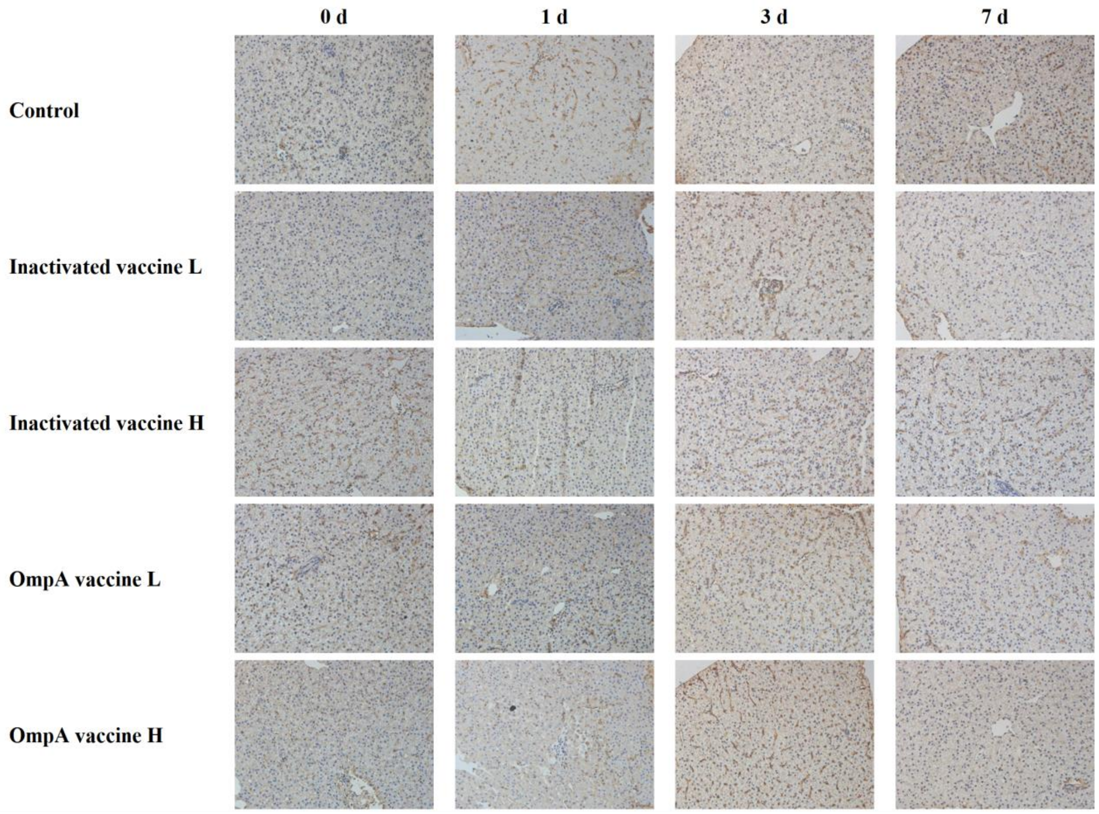

Supplemental Fig. 5C. Positive cells to anti-IgM antibody in the hepatopancreas that detected by IHC.

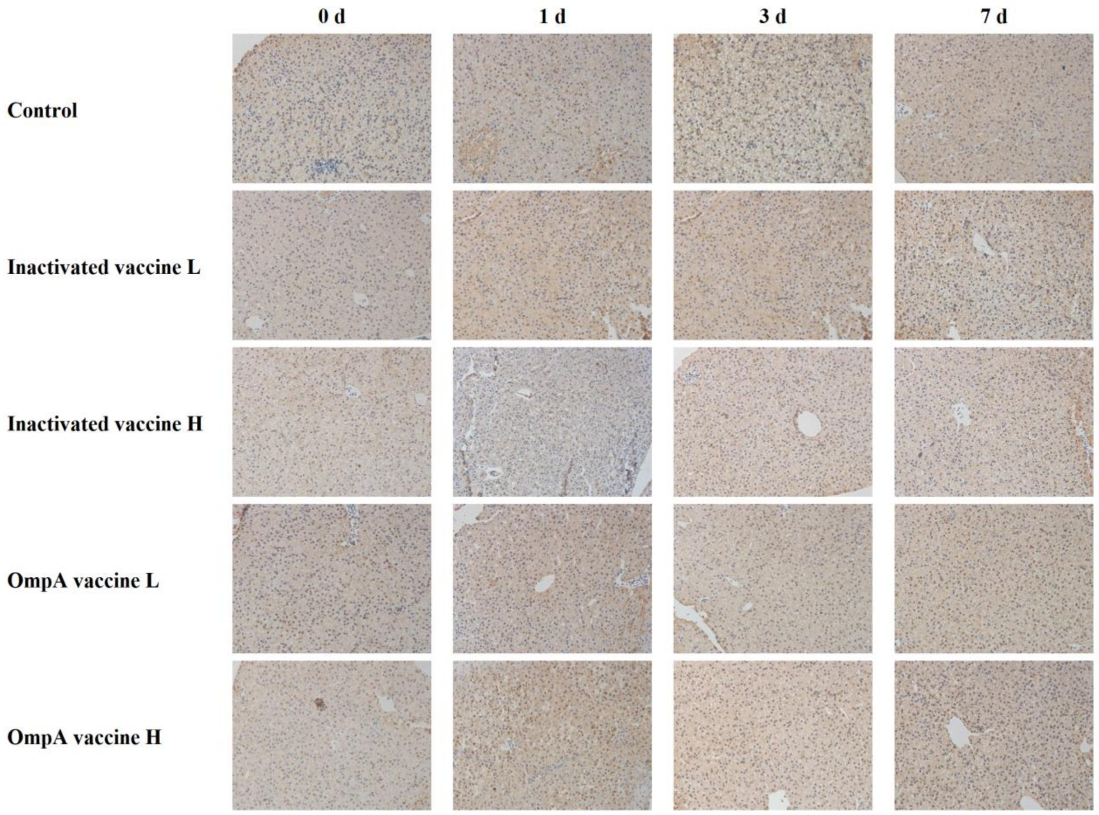

Supplemental Fig. 5D. Positive cells to anti-IgZ antibody in the hepatopancreas that detected by IHC.

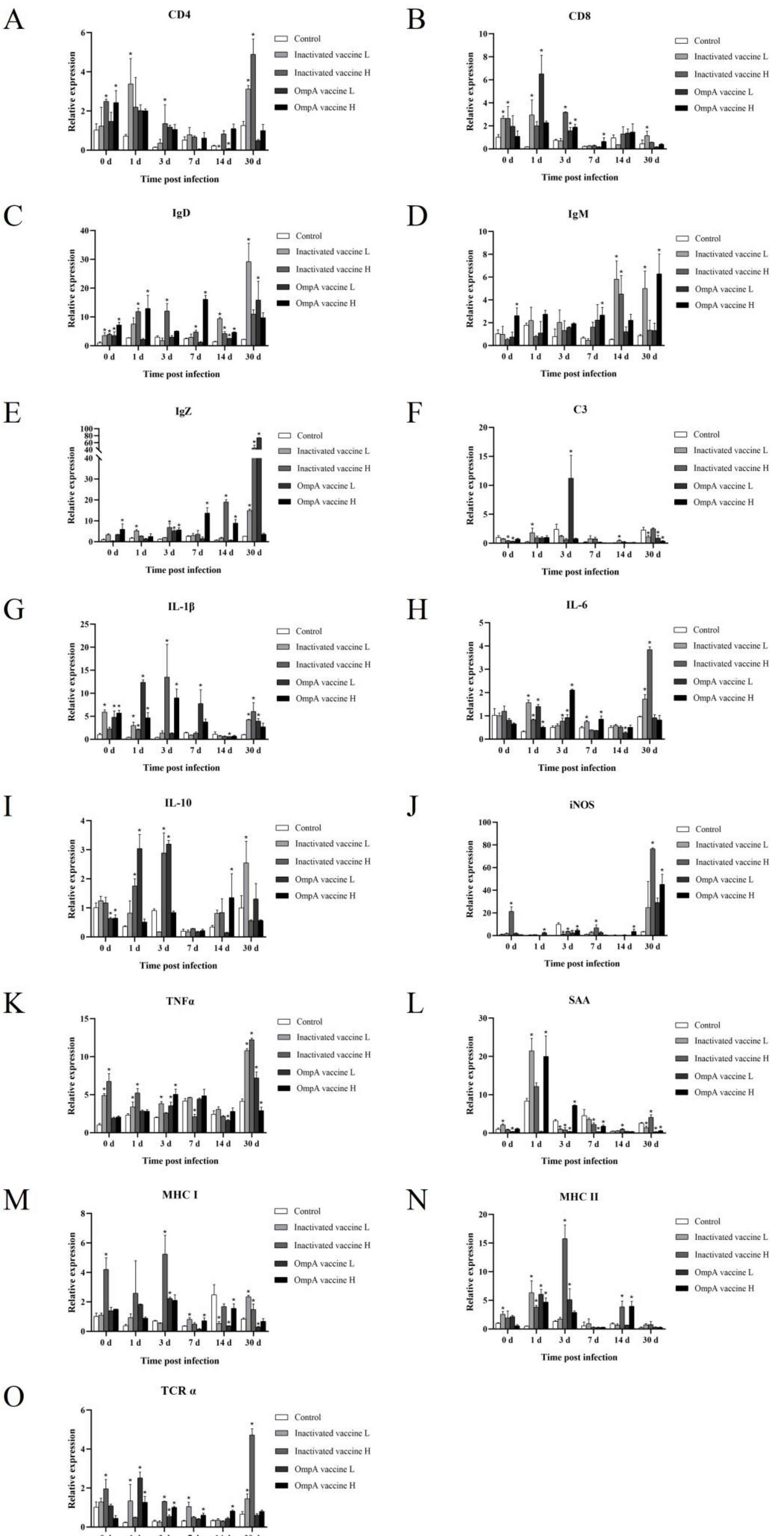

Supplemental Fig. 6A. Expression of immune related genes in the intestines of *M. amblycephala*.

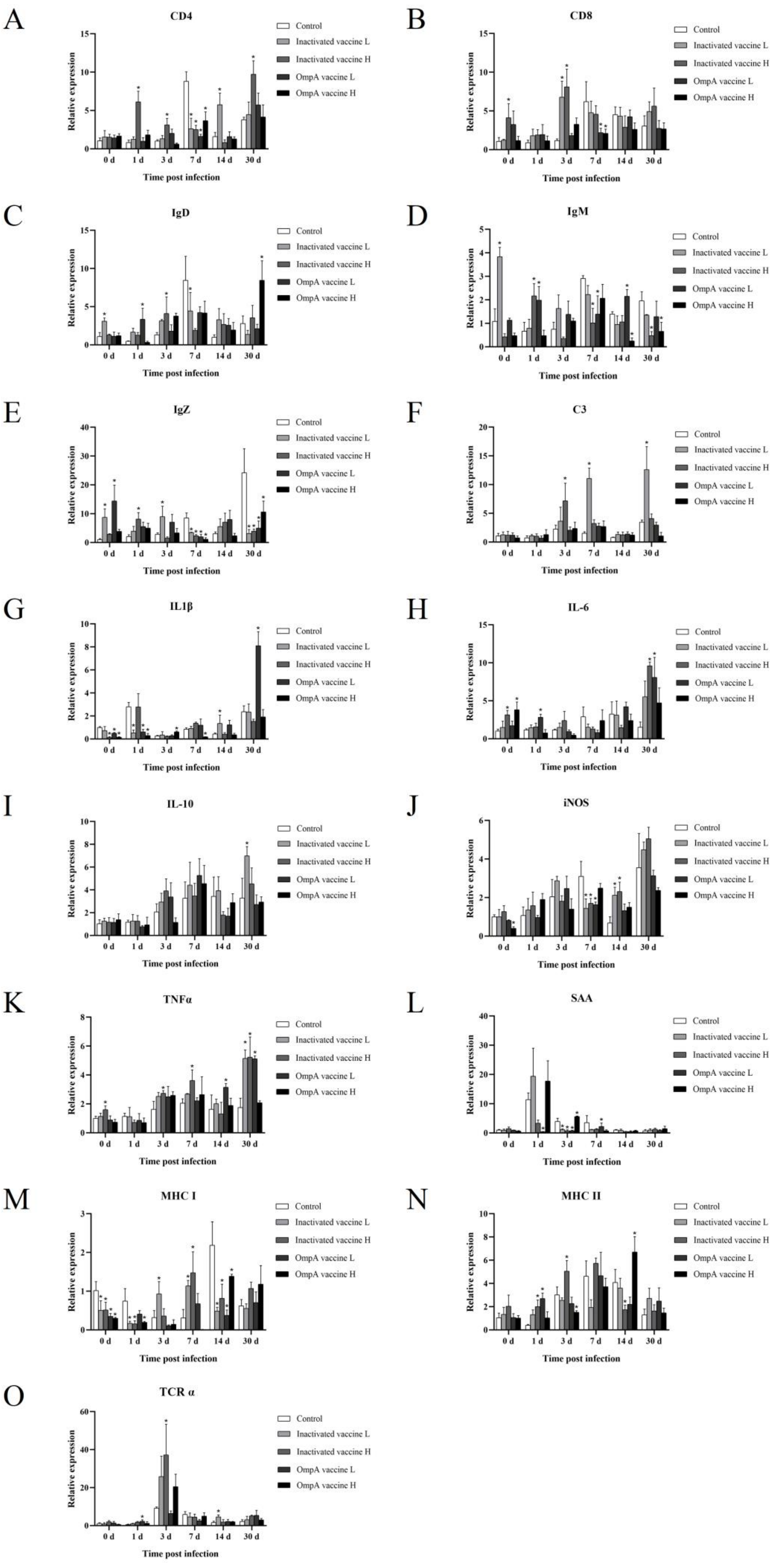

Supplemental Fig. 6B. Expression of immune related genes in the gills of *M. amblycephala*.

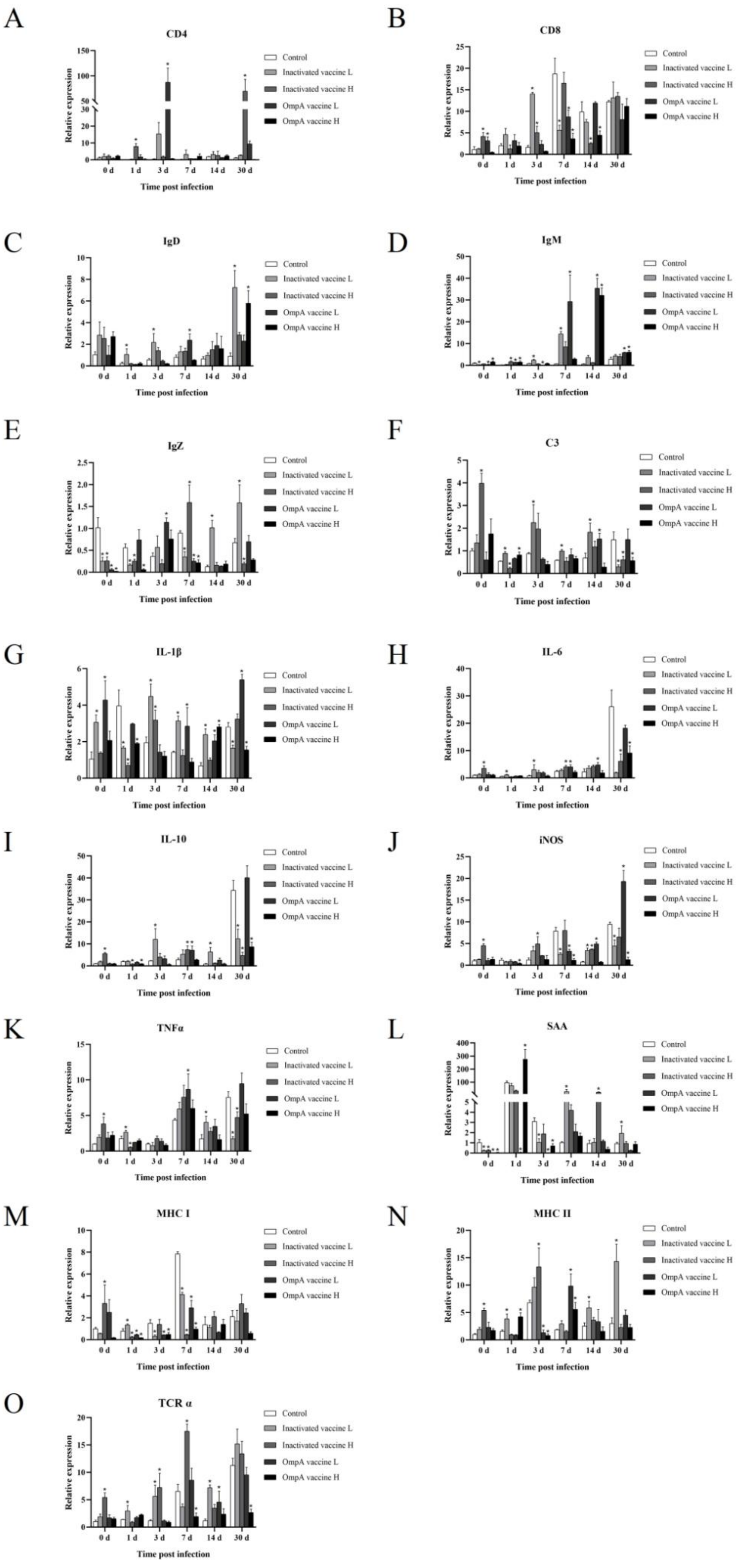

Supplemental Fig. 6C. Expression of immune related genes in the hepatopancreas of *M. amblycephala*.

Supplemental Table 1. Primers used for PCR in the present study.

**Primers**	**Sequences (5'-3')**
OmpA-F	CCATGGCTGACGACATCTACTTCGGTGCC
OmpA-R	CTCGAGCTTCTGAACTTCTTGTACGCCAGA
q*SAA*-F	GTGCTGGTGCTGGGTTTGGT
q*SAA*-R	GCCTTCCTCATATCCTGGTAGGC
q*iNOS*-F	ATTCAAGGGCAGCTTCCAGG
q*iNOS*-R	CAGGGGCAAAGTTTAAGGGC
q*IL1β*-F	CGATAAGACCAGCACGACCTT
q*IL1β*-R	GTTTCCGTCTCTCAGCGTCA
q*IL6*-F	AAGACAACCGCACACTCGAT
q*IL6*-R	CTGGGTCTCTTCACGCCTTT
q*IL10*-F	GTGTTTTCGGGTGCAAGTGG
q*IL10*-R	ATGAACGAGATCCTGCGCTT
q*TNFα*-F	TGATGACGGCATTTACTTCG
q*TNFα*-R	CCTCCATAGGAATCAGAATAGC
q*C3*-F	ATGGACTTTCACTCGATCCAACA
q*C3*-R	AACTGCTTCTCCATCTTCACACT
q*MHC I*-F	TTCCAGACGACGAGCACCAT
q*MHC I*-R	ACACCACATACCCAGCGACA
q*MHC IIβ*-F	CTTCTACCCACCCCAGATCC
q*MHC IIβ*-R	GTGATCCACAGCACAGGAGA
q*CD4*-F	GAGACGAATTTGATGGTTGTGGC
q*CD4*-R	GCTGCTTTGATTGCTGGGAAGT
q*CD8α*-F	TTCACTGCGACCCTAAACCG
q*CD8α*-R	TTAATGTCCAAGCGTACCTTACCA
q*TCRα*-F	TGGCAACTGACTTCACGAAC
q*TCRα*-R	CAAAAGAGCCCCAGTGACAT
q*IgM*-F	GGAGCAACGGCACAGTAT
q*IgM*-R	ATCAGCAAGCCAAGACAC
q*IgD*-F	TGGCTGCTTGGAACGACT
q*IgD*-R	ATGTTATGCGACTGGGTA
q*IgZ*-F	CCAAAGTAAACCGAAGTG
q*IgZ*-R	AAAGTGATAGAGGCAGAAC
q*16S rRNA*-F	GGGAGTGCCTTCGGGAATCAGA
q*16S rRNA*-R	TCACCGCAACATTCTGATTTG
q*GAPDH*-F	TGCCGGCATCTCCCTCAA
q*GAPDH*-R	TCAGCAACACGGTGGCTGTAG
